# Factors related to axial length elongation and myopia progression in orthokeratology practice

**DOI:** 10.1371/journal.pone.0175913

**Published:** 2017-04-18

**Authors:** Bingjie Wang, Rajeev K. Naidu, Xiaomei Qu

**Affiliations:** 1 Eye & ENT Hospital, Fudan University, Shanghai, China; 2 School of Medicine, University of Sydney, Camperdown, NSW, Australia; 3 Key Laboratory of Myopia, Ministry of Health, Fudan University, Shanghai, China; Seoul National University College of Pharmacy, REPUBLIC OF KOREA

## Abstract

**Purpose:**

To investigate which baseline factors are predictive for axial length growth over an average period of 2.5 years in a group of children wearing orthokeratology (OK) contact lenses.

**Methods:**

In this retrospective study, the clinical records of 249 new OK wearers between January 2012 and December 2013 from the contact lens clinic at the Eye and ENT Hospital of Fudan University were reviewed. The primary outcome measure was axial length change from baseline to the time of review (July-August 2015). Independent variables included baseline measures of age at initiation of OK wear, gender, refractive error (spherical equivalent), astigmatism, average keratometry, corneal toricity, central corneal thickness, white-to-white corneal diameter, pupil size, corneal topography eccentricity value (e-value), intraocular pressure (IOP) and total time in follow-up (months total). The contributions of all independent variables on axial length change at the time of review were assessed using univariate and multivariable regression analyses.

**Results:**

Univariate analyses of the right eyes of 249 OK patients showed that smaller increases in axial length were associated with older age at the onset of OK lens wear, greater baseline spherical equivalent myopic refractive error, less time in follow-up and a smaller e-value. Multivariable analyses of the significant right eye variables showed that the factors associated with smaller axial length growth were older age at the onset of OK lens wear (p<0.0001), greater baseline spherical equivalent myopic refractive error (p = 0.0046) and less time in follow-up (p<0.0001).

**Conclusions:**

The baseline factors demonstrating the greatest correlation with reduced axial length elongation during OK lens wear in myopic children included greater baseline spherical equivalent myopic refractive error and older age at the onset of OK lens wear.

## Introduction

Myopia is one of the leading causes of visual impairment and vision loss worldwide [1, 2]. With its increasing burden of disease and varying prevalence rates amongst different ethnic groups, identifying those most at risk of myopia progression is becoming increasingly more important. Over the past half century, East Asia has seen a significant rise in the incidence of myopia, particularly in children [3, 4], with prevalence rates of up to 90% in Taiwanese University students [5]. A recent meta-analysis projected that myopia and high myopia will affect 5 billion and 1 billion people, respectively, by the year 2050 [2].

Although the exact aetiology of myopia is still not clearly understood, it typically develops from around the age of 8 years old and is known to be related to an increase in the axial length of the eye [6, 7]. This growth greatly increases the risk of a number of potentially sight-threatening ocular complications, such as cataract, glaucoma, staphyloma and retinal complications [8, 9]. With myopia becoming an increasing public health problem, the challenge of how to control the development of myopia has become an important area of research.

There are a number of pharmacological and optical treatment methods that have been used to help slow the progression of myopia [1, 10–13]. Current evidence suggests that medications such as 0.01% atropine are effective agents in reducing myopia progression; however, the effects of the long-term use of anti-muscarinic drugs in children remain to be elucidated [14]. Orthokeratology (OK) is an optical treatment option using specially designed reverse geometry contact lenses that are worn overnight [11, 15]. The OK lens reshapes the corneal surface during sleep to attain refractive error correction, with this effect lasting throughout the following day. Therefore, one of the major advantages of OK over atropine therapy is that it allows for good vision throughout the day without the need for optical correction.

OK has also become a popular treatment option due to reports of its ability to safely control myopia progression in children [15–19]. Currently, OK has been shown to be the most effective non-pharmacological treatment option for slowing myopia progression, with several researchers demonstrating a similar efficacy to that of atropine [13, 16, 17, 19–22]. Recent studies investigating the effects of different factors, such as initial age, baseline myopia, age of myopia onset and parental refraction, on the efficacy of OK on myopia progression, however, have reported contradicting results, making it difficult to predict which children are likely to gain the most benefit from OK wear [16, 17, 23, 24].

A number of studies comparing OK to other treatment modalities have found that those with a greater degree of baseline myopia experienced less myopic progression [23, 25]. Cho and Cheung reported that older children tended to have reduced axial length elongation, for both OK and spectacle-wearing children, although baseline myopia had no effect on myopia progression [16]. Santodomingo-Rubido *et al*. evaluated factors that may contribute to axial length change in OK versus spectacle wearers over a course of 2 years, concluding that older age and greater central corneal power were associated with smaller axial length elongation, but that baseline myopia demonstrated no significant relationship with axial length elongation in OK wearers when compared to spectacle wearers, in either univariate or multivariate analyses [24].

Several studies exist demonstrating the efficacy of OK in controlling myopia progression compared to other forms of refractive corrections; however, identifying the children who are likely to gain the greatest benefit from myopic control with OK specifically is a big clinical challenge. To date, no long-term, large-scale study exists in the literature investigating the relationships of different patient baseline factors on axial length elongation in children wearing OK lenses. Therefore, the purpose of this study was to evaluate the degree to which several baseline and treatment duration factors contribute to axial length growth and myopia progression in a larger sample of children corrected with OK lenses over an average of 2.5 years of OK lens wear.

## Materials and methods

### Subjects

The clinical records of new OK contact lens patients who presented to the Contact Lens Clinic at the Eye and ENT Hospital of Fudan University between January 2012 and December 2013 were reviewed. In total, 249 subjects were deemed suitable for this study and included for analysis according to the inclusion criteria below (Table 1). Only data from the right eye was used for statistical analysis. This study was approved by the Institutional Review Board of the Ethics Committee of the Eye and ENT Hospital of Fudan University—Approval Number 2014042–2. This study was conducted in accordance with the Tenets of the Declaration of Helsinki. Written informed consent was obtained from the next of kin, caretakers, or guardians on behalf of the minors/children at the time of admission to the Eye & ENT Hospital of Fudan University. Data was collected from the hospital records and no patient involvement was required.

**Table 1:** Inclusion criteria for data collection.

Inclusion criteria
1. Age: between 7–15 years. 2. No prior history of contact lens or OK wear. 3. Best corrected visual acuity of logMAR 0.1 or better. 4. At least -0.75DS of myopia in one eye, and no more than -1.00DS of myopia difference between both eyes. 5. Ocular health status for suitability for OK wear was screened using pre-wear corneal topography. 6. Unaided visual acuity of logMAR 0.1 or better at the last scheduled review appointment. 7. Subjects maintained regular follow-up appointments and was still being reviewed at the clinic at the time of review for this study. 8. Subjects had worn lenses for at least 1 year at the time of review.

OK = Orthokeratology contact lenses, logMAR = log Minimal Angle of Resolution, DS = Diopters of Sphere.

### Data collection

Data was retrospectively collected from the clinical records of the 249 subjects, only the right eye data (total 249 eyes) was used for statistical analysis. The data collected included: age (in years) at the initial OK lens fitting, gender, baseline spherical equivalent (SE) refractive error (SE = spherical power + ½ cylindrical power), keratometry readings (from corneal topography), intraocular pressure (IOP), corneal topography eccentricity value (e-value), and total length of OK wear (time spent in follow-up = time from the first day of initiating OK wear to the latest axial length measurement, recorded as months) as detailed below.

### Clinical assessment

All patients underwent a standard anterior eye and refractive status assessment prior to commencing OK wear. This assessment included a measurement of baseline corneal topography using the Oculus Pentacam system (Oculus, Wetzlar, Germany). The mean corneal power was calculated by averaging the flatter and steeper keratometry values. The difference between the flat and steep keratometry values was calculated as total corneal toricity. The central corneal thickness (CCT) and corneal e-values were also obtained from the Oculus Pentacam system.

A subjective cycloplegic refraction was performed after the instillation of three drops of 0.5% tropicamide (Santen Pharmaceutical Co. Ltd. China), separated 5 minutes apart in each eye, to measure the baseline myopic refractive error in each patient. Axial length and white-to-white corneal diameter (WTW) were measured using the Zeiss IOL Master (Carl Zeiss Jena GmbH, Jena, Germany). Three separate measurements were performed in total, and the average value was recorded.

### Schedule of visits

All patients were followed-up according to the standard protocol of the Contact Lens Clinic at the Eye and ENT Hospital of Fudan University. Patients were typically reviewed at 1 day, 1 week and 1 month following OK wear. Follow-up appointments were thereafter scheduled every 3 or 6 months. At each visit, measurements of visual acuity using a Snellen chart, and slit-lamp examinations assessing ocular health and OK lens integrity were performed. Axial length was measured at different review intervals deemed suitable by the clinician, however due to the nature of the contact lens clinic at the hospital, axial length measurements were measured at differing intervals. As heterogeneity existed in axial length measurement intervals amongst a large clinical setting, only the most recent axial length measurement was obtained and the number of months of OK wearing time up until that point was recorded. An average 12 months axial length change was then calculated by subtracting the most recent axial length from the baseline value and divided by the total months of OK wear, then times 12. All patients had no more than 30 days of lens cessation during the review period.

### Statistical analysis

The primary outcome measure of this study was the change in axial length from baseline to the time of review (July 2015—August 2015). Independent variables of the baseline data included: age at initiation of OK lens wear (years), gender, SE refractive error, average corneal power, corneal toricity (calculated as the difference between the flat and steep keratometry values), CCT, WTW diameter, pupil size, corneal e-value, IOP and total OK wearing time (time in follow-up; months total). The contribution of each of the independent variables on axial length change at the time of review was assessed using simple linear regression for the Right eyes of all subjects. Factors that had a statistical significance level of p<0.05 were selected to enter into the multivariable regression analysis model using the backward stepwise removal method. The strength of association for significant variables is represented using beta values, 95% confidence intervals, corrected R^2^ values and p values.

## Results

### Patient and treatment characteristics

Of the 249 subjects, there were 137 females and 112 males. The average age at the initiation of OK lens wear was 9.75±1.96 years old and the average duration of OK lens wear was 29.62±6.64 months. The average baseline SE refractive error was -3.03±1.11 Diopters of Sphere (DS) and the average amount of axial elongation was 0.21±0.15 mm/year. (Table 2)

**Table 2:** Univariate regression analyses of different independent variables on axial length elongation.

Variable	Value (Mean)	B value	R^2^	P value	95% Confidence interval	
**Gender**	F: 137, M: 112	0.08000	0.0116	0.0905	-0.01271	0.17271
**Baseline age (years)**	9.75±1.96	-0.10184	0.2904	<0.0001*	-0.12180	-0.08189
**SE (DS)**	-3.03±1.11	0.08073	0.0586	<0.0001*	0.04017	0.12129
**Corneal power (DS)**	43.28±1.04	-0.00734	0.0004	0.7453	-0.05182	0.03713
**Corneal toricity (DC)**	1.01±0.42	-0.01297	0.0002	0.8169	-0.12322	0.09727
**CCT (μm)**	554.00±30.51	-0.00114	0.0087	0.1425	-0.00266	0.0003854
**WTW (mm)**	11.69±0.32	-0.04398	0.0014	0.5569	-0.19124	0.10328
**Duration (months)**	29.62±6.64	0.01078	0.0373	0.0022*	0.00391	0.01764
**Pupil size (mm)**	3.87±0.56	0.00194	0	0.9635	-0.08152	0.08541
**E-value**	0.54±0.12	0.49712	0.0266	0.0100*	0.11997	0.87427
**IOP (mmHg)**	15.69±2.69	-0.00897	0.0042	0.3065	-0.02620	0.00827

SE = Spherical equivalent, DS = Diopters of Sphere, DC = Diopters of Cylinder, CCT = Central corneal thickness, WTW = white-to-white corneal diameter, E-value = corneal eccentricity value, IOP = intraocular pressure, mmHg = millimeters of Mercury.

* p<0.05 = Statistically significant.

### Univariate analysis

Linear regression analyses of the independent variables in the right eyes of all subjects showed statistically significant associations between age at initiation of OK wear, baseline myopic refractive error, corneal e-value and duration of lens wear with axial length change. Univariate analyses of the right eyes of the 249 OK patients showed that smaller increases in axial length were associated with older baseline age at the onset of OK lens wear, greater baseline spherical equivalent myopic refractive error, less time in follow-up and a smaller e-value.

**Fig 1:**
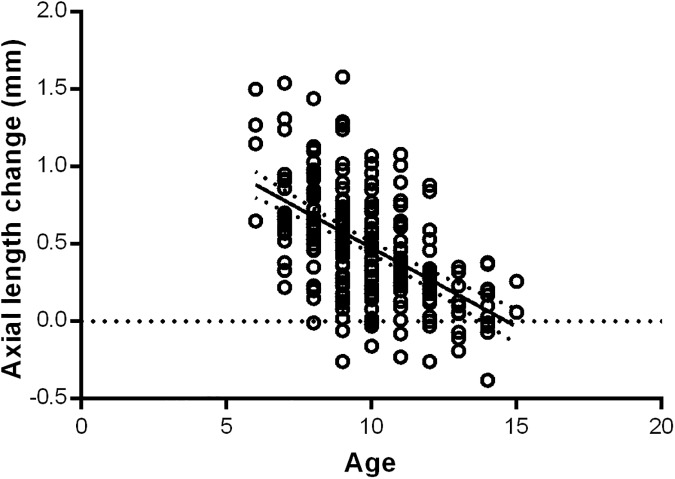
Simple linear regression between axial length change and baseline age of OK wear.

The older the baseline age at the time of initiation of OK lens wear, the smaller the axial length elongation over the course of the patient’s review period (R^2^ = 0.2904, p<0.0001, Fig 1). Baseline age displayed the strongest relationship with axial length change of all the independent variables.

**Fig 2:**
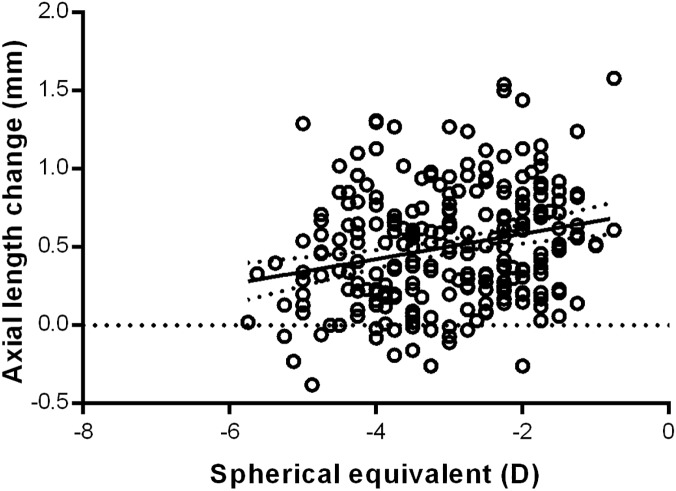
Simple linear regression between axial length change and baseline SE refractive error (DS).

Subjects with a greater baseline SE refractive error, demonstrated smaller increases in axial length elongation during the duration of lens wear (R^2^ = 0.0586, p<0.0001, Fig 2). Therefore, those with a greater degree of myopia experienced a smaller change in axial length over the duration of the review period.

**Fig 3:**
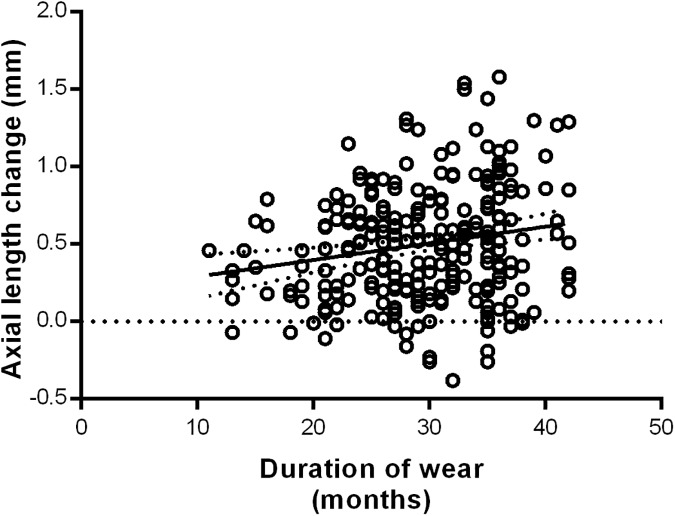
Simple linear regression between axial length change and duration of lens wear (months).

Subjects who spent less time in follow-up demonstrated a smaller degree of axial length elongation (R^2^ = 0.0373, p = 0.02, Fig 3). The length of OK lens wear of subjects in this study ranged from a duration of 12 to 42 months, with those who had been followed-up for a shorter duration of time experiencing a smaller change in axial length.

**Fig 4:**
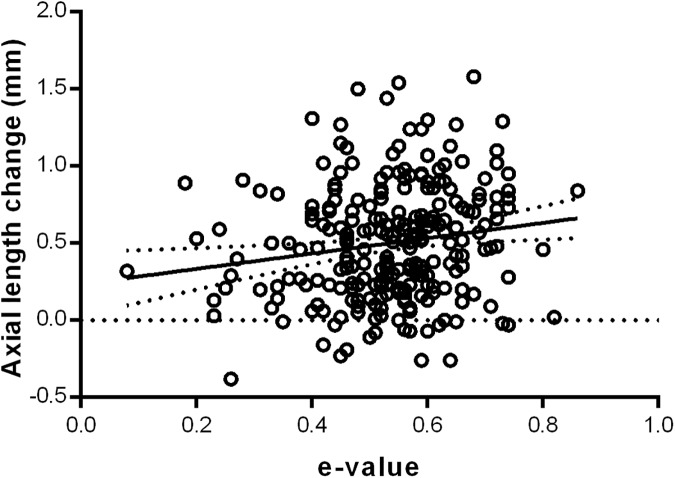
Simple linear regression between axial length change and E value.

Baseline corneal eccentricity demonstrated a statistically significant relationship with axial length change, with a greater rate of peripheral corneal flattening (greater e-value) associated with a greater degree of axial length change (R^2^ = 0.0226, p = 0.01, Fig 4). Therefore, the more prolate the cornea (greater e-value), the greater the axial length elongation, while those with steeper peripheral corneas experienced a smaller change in axial length. Linear regression analyses of the other independent variables showed no statistically significant relationships between them and axial length progression. Therefore, the results of the present study demonstrated no significant associations between gender, mean corneal power, corneal toricity, CCT, WTW diameter and pupil size with axial length elongation after OK lens wear (Table 2).

### Multivariable analysis

Multivariable analyses of the right eye data showed that the factors associated with smaller increases in axial length were older baseline age at initiation of OK lens wear (p<0.0001), greater baseline spherical equivalent myopic refractive error (p = 0.0046) and less time in follow-up (p<0.0001). Although there appeared to be an association between baseline corneal e-value and axial length growth in univariate analyses, multivariable analyses did not show any statistically significant relationship between the e-value and axial length growth after OK lens wear (Table 3).

**Table 3:** Multivariable regression analysis showing the strength of association between the independent variables and axial length growth.

Variable	B value	P value	95% Confidence Interval	
**Age (years)**	-0.09736	<0.0001*	-0.11688	-0.07784
**SE (SD)**	0.07113	0.0046*	0.02219	0.12007
**Duration (months)**	0.01194	<0.0001*	0.00627	0.01760
**E value**	0.18602	0.2415	-0.12608	0.49812
**Final model**	R^2^ = 0.3669	<0.01		

SE = Spherical equivalent, DS = Diopters of Sphere, E-value = corneal eccentricity value.

* p<0.05 = Statistically significant.

## Discussion

With the increasing visual demand in today’s modernized society, myopia is becoming a more prevalent cause of reduced vision and a greater public health issue globally [3, 26, 27]. Due to its increasing prevalence and growing disease burden, there is now a greater need for a better understanding of a treatment modality that can help slow this trend. Slowing the progression of myopia will likely afford a great benefit to a large number of people, especially children in Asian countries, where the emphasis on academic performance and increased near visual demand have led to the highest prevalence of myopia and high myopia worldwide [3, 4, 8, 10, 26–29]. Many myopic treatment options, such as spectacle and contact lens correction and corneal refractive surgery, simply correct the refractive error, but have little impact on the progression of myopia itself [1]. In order to prevent the complications associated with myopia progression and high myopia, the ideal treatment modality would be one that not only corrected the refractive error, but also halted the progression of myopia.

Several studies in recent years have now demonstrated the benefit of OK over other treatment modalities in assisting to reduce axial length growth and thereby slow the rate of myopia progression in children [15–18, 20, 21, 23, 28, 30, 31]. Many of these studies, however, have investigated the effect of OK on differing populations with varying baseline factors and treatment regimes. Also, they have often studied small sample sizes, with follow-up periods limited to 2 years, with the first long-term study, conducted by Hiraoka *et al*., comparing axial length elongation in only 59 myopic children receiving OK versus spectacle treatment over a period of 5 years [17]. Therefore, the present study was conducted to evaluate the degree to which several baseline and treatment duration factors contribute to axial length growth and myopia progression in a larger sample of children corrected with OK lenses over and an average of 2.5 years of OK lens wear.

Despite its increasing prevalence, the risk factors that lead to the progression of myopia are still not fully known and the clinical challenge of identifying which patients are most suitable for OK treatment to control myopia progression is a big one [12]. A number of different patient factors have been identified as having the potential to affect the rate of myopia progression; however, several studies have provided contradicting results on the relationships between these factors and axial length growth [16, 17, 23, 24, 28, 30]. A better understanding of these relationships may help predict those patients that are likely to gain the most benefit from OK. In the present study, we investigated the relationships between a number of different baseline patient factors and axial length change after OK lens wear. All of these factors are measurements and data that can be easily obtained in a clinical setting when assessing and fitting patients with OK. One of the main aims of this study was to evaluate which of these factors may help better identify the patients who are likely to experience the greatest amount of axial length change and myopia progression, and thereby gain the greatest benefit from early OK lens wear to prevent this progression.

OK has now been proven to be an effective treatment option in controlling axial length growth and myopia progression in children [15, 16, 23, 28, 31]. No comparison was made in this study between other treatment modalities on axial length change, such as pharmacological or spectacle lens treatments, with the primary outcome measure of interest being the amount of change in axial length from baseline to the time of review of this study. The results of the present study demonstrated statistically significant relationships between age at initiation of OK wear, baseline myopic refractive error and duration of lens wear with axial length change. We demonstrated no significant associations between gender, mean corneal power, corneal toricity, CCT, WTW diameter, pupil size or IOP with axial length elongation after OK lens wear, suggesting that these physical attributes of an individual’s cornea do not appear to affect the myopic control effect of OK wear.

Baseline SE refractive error may provide a predictive factor in axial length change in myopic children corrected with OK, however previous studies have generated conflicting results in regards to its relationship with axial length change [16, 17, 20, 23–25, 28, 32]. We found that a higher baseline myopia was significantly associated with a reduced amount of axial length change following OK wear (R^2^ = 0.0586, p<0.01, Fig 2). This is in agreement with a number of previous studies that have reported greater benefits from OK in children with higher myopia, with reduced efficacy of OK in low myopia compared to high myopia [17, 25, 28]. One proposed theory for this effect is that there is a greater degree of corneal steepening in the mid-periphery of higher myopic eyes, thereby having a greater beneficial impact on the peripheral retinal defocus and thus further slowing the stimulus for axial length growth and myopia progression [20, 31]. There may also be a greater degree of myopia progression in the early stages, with a natural slowing of axial length growth once the eye is nearing a potential myopic and axial length threshold for that individual.

A select few studies, however, have shown either no relationship or the opposite relationship between baseline myopia and axial length change [16, 21, 23, 24]. Santodomingo-Rubido *et al*., who examined the predictive factors for axial length growth over 2 years of OK lens wear, showed no significant relationship between baseline myopia and axial length change [24]. Their results of 31 children wearing OK lenses, however, suggested the possibility of reduced axial length change with lower degrees of baseline myopia; although due to their small sample size, their study may not have been sufficiently powered to detect a difference. This discrepancy may, in part, be due to different lens designs that have different effects on corneal shape and peripheral defocus [33]. However, the majority of studies still conclude that there is a greater benefit in reducing axial length change in children with higher baseline myopia, which our results confirm. At present, it is still unclear what the exact relationship is between baseline SE refractive error, the degree of peripheral defocus and axial length progression, and further studies with longer follow-up periods are required to ascertain the answer to this question.

The age of patients suitable for and fitted with OK lenses is an important consideration, as the clinical benefits of earlier reductions in axial length growth and myopia progression with the use of OK lenses needs to be balanced against the safety and practical limitations of rigid gas permeable contact lens wear in young children. Our results indicated that children that were older at the time of initiation of OK wear experienced less axial length growth than those of a younger age (R^2^ = 0.2904, p<0.01, Fig 1). This is in agreement with the findings of a recent study by Santodomingo-Rubido *et al*., who also concluded that the older the age at baseline, the smaller the axial elongation after 2 years of OK wear, despite their results not being statistically significant [24]. It is difficult to determine whether this effect is due to the refractive effect of OK or simply due to the natural progression and development of myopia. Children that are of older age and with a later onset of myopia may already be experiencing a natural reduction in the rate of axial elongation [6, 17, 29]. This may help to further justify the conclusions of a number of previous studies that showed greater myopia control benefit in younger patients, whereby, this difference may be better explained by the effect of OK on slowing the progression of faster rates of axial length elongation in younger children [16]. The ROMIO study suggested that baseline age may reflect the rate of myopia progression, with younger children shown to undergo faster rates of axial elongation, which has also been proposed by others previously [4, 16, 29]. The use of OK in this younger group of children helped to reduce the proportion of children who developed high myopia quickly due to the slowing of their faster axial elongation. Therefore, because younger children may experience greater and faster axial length growth, they may gain the most benefit from early intervention with OK, to help slow the progression of myopia earlier on, thus reducing the prevalence of high myopia.

Corneal eccentricity was shown to have a statistically significant relationship with axial length change (R^2^ = 0.0226, p = 0.01) in univariate, but not multivariable analyses. We found that a greater corneal e-value, which represents a more prolate or flatter peripheral cornea, was associated with a greater change in axial length (Fig 4). A more prolate peripheral cornea results in greater peripheral retinal hyperopic defocus, which is thought to drive axial length growth. OK works to correct myopic refractive error by flattening the central cornea and steepening the mid-peripheral cornea, with a better fitting OK lens capable of achieving a more accurate and controlled steepening of the mid-peripheral cornea, thereby helping to better control the progression of myopia [30, 34, 35]. We propose that a steeper peripheral cornea may allow for better lens stabilisation, which is largely driven by the peripheral cornea-lens relationship or lens sagittal height, and thus result in greater peripheral refractive correction. However, as corneal eccentricity was only found to be associated with axial length change in univariate analyses and not in multivariable analyses, it may not in fact play a role in axial length change with OK wear. This, however, may be due to a confounding of its association by another factor (or factors), causing attenuation of the association between corneal eccentricity and myopia progression in the multivariable analysis.

The effect of OK lens wear on controlling myopia progression has been shown to be time-dependent [16, 17, 19, 23, 28, 31]. Our results indicated that subjects who spent less time in follow-up demonstrated a smaller degree of axial length elongation (R^2^ = 0.0373, p = 0.02, Fig 3). The length of OK lens wear of subjects in this study ranged from a duration of 12 to 42 months, with those who had been followed-up for a shorter duration of time experiencing a smaller change in axial length. This may however, simply be due to the fact that these patients were followed-up over a shorter period of time, having less time for axial length growth, and may not necessarily reflect the myopia control effect of OK. We were also unable to determine whether or not there was any change in the rate of axial length growth over the various periods of OK wear. Our results on the effect of duration of OK lens wear on axial length growth, therefore, do not provide any further evidence on the control effect of OK, and would require a control group for comparison in order to make any definitive conclusions.

Many previous studies have investigated the effect of OK over a fixed period of time, typically 2 years of OK wear, with many concluding that the effect of OK on myopic control was most noticeable in and largely limited to the first 12 to 18 months of wear [6, 17, 19, 23, 28, 29]. Participants in our study demonstrated a similar amount of axial length change over 12 months as those wearing OK in previous studies, with the average calculated axial length change in our subjects being 0.21±0.15mm per year. Wen *et al*. conducted a systematic review and meta-analysis of studies investigating the comparative outcomes of the efficacy and acceptability of OK wear in myopic children, with axial length change the primary outcome measure [31]. They reported a similar average axial length change in the OK groups of the included studies, with all groups showing significantly less axial length compared to the control groups in each study. They also reported a gradual reduction in the myopia control rate over time, with rates of 55, 51, 51 and 41% recorded for 6, 12, 18 and 24 months respectively, following OK wear compared to controls after 2 years of follow-up. In a 5 year follow-up study by Hiraoka *et al*., they too reported no additional benefit of OK on the control of myopia progression after 3 years of OK lens wear; proposing that changes in axial length after this time are more difficult to detect due to the natural slowing of axial elongation with time [17]. Also, the possibility of a rebound phenomenon in myopia progression after discontinuation of OK wear, such as that observed after the discontinuation of atropine [36], is still unknown, and further studies looking at the longer term effects of OK, including after cessation of lens wear, are needed.

The accuracy, reliability and repeatability of axial length measurements are critical in assessing the efficacy of OK in slowing myopia progression. In the present study, axial length was evaluated by non-contact optical biometry with the use of the IOLMaster. Earlier studies used classical ultrasound A-scan devices to measure axial length in their OK patients, however, this is a contact technique that may prove difficult and unreliable in children [19, 28]. Many more recent studies now use the non-contact IOLMaster for measurements of axial length due to its high reproducibility, precision, speed and ease of use [16, 17, 20, 21, 23, 25, 30]. The accuracy of its readings has also been shown to be equivalent to that of ultrasound A-scan measurements [37], with Wen *et al*. finding no significant differences in the efficacy of the IOLMaster in measuring axial length when compared to A-Scan measurements [31]. For these reasons, the IOLMaster appears to be an accurate and more suitable measure of axial length, particularly in children wearing OK lenses.

The present study has a number of strengths that allows our findings to add to the current literature. To our knowledge, this study is the first of its kind to include such a large sample size (n = 249) of children wearing OK lenses for the control of myopia. This large sample size allows greater statistical power to detect any significant relationships between the various factors and axial length in myopia progression. In addition to the large sample size, there was relative homogeneity amongst the subject variables.

This study also had a number of other limitations. It was a retrospective study, but had no control group to compare the results of the OK-wearing children to. We also investigated a limited range of myopia, and including subjects with much higher degrees of myopia may provide further insight into the control effect of OK in highly myopic patients. Axial length was also measured over a range of different time intervals and averaged for the different subjects, which may not have allowed us to detect more subtle changes in axial length amongst certain subjects; however, this was factored in to the analyses by looking at time as an independent variable as well.

In the present study, the range of follow-up varied between 12 to 42 months. Unfortunately, this was a weakness of the study due to the busy nature of the clinical setting of the hospital, and thus not all patients could be reviewed consistently on the same regular review schedule (for example exactly every 6 or 12 months). Because of the variability in the length and intervals of the axial length measurements, it was not possible to record 12- or 24-monthly average axial measurements. Also, a number of other factors that may be of particular relevance were not included in the measurements and analyses. These included anterior chamber depth, age of myopia onset, myopia progression 2 years prior to OK wear and parental refractions. Although not all of these factors are readily assessable in a clinical setting, any of these factors may have demonstrated a significant relationship with axial length elongation and further investigations into their effects should be conducted. Finally, we were unable to determine the optimal treatment duration for OK wear to completely halt axial length change and myopia progression, and further long term studies looking at this are needed to determine whether the effect of OK is sustained and when it is safe to cease lens wear.

## Conclusions

It has been shown in previous studies that OK is an effective optical treatment option to help slow axial length growth and myopia progression in children with myopia. Our study found that a number of baseline factors exist that demonstrate a significant relationship with axial length change [15–20]. The baseline factors demonstrating the greatest correlation with reduced axial length elongation after OK lens wear included greater baseline spherical equivalent myopic refractive error and older age at the onset of OK wear. Therefore, in a clinical setting, the assessment of baseline parameters, such as age and degree of refractive error, are important in screening and prognosticating which patients are likely to gain the most benefit from OK lens wear; with those that demonstrate greater degrees of axial length change and myopia progression, particularly younger children and SE refractive errors closer to emmetropia, being likely to gain the most long-term benefit from earlier myopia control using OK. Further studies are required to determine whether the impact of OK on these modifiable and variable baseline factors has a true effect on axial length growth and myopia progression, or whether they are independent of OK wear.

## Supporting information

S1 Dataset Raw data table. (PDF)
