# Supplementary material for: Factors related to axial length elongation and myopia progression in orthokeratology practice
Source: PLoS One. 2017 Apr 18;12(4):e0175913. doi: 10.1371/journal.pone.0175913 (PMC5395222; doi:10.1371/journal.pone.0175913)
Supplement: S1 Dataset — (PDF) [file pone.0175913.s001.pdf]

Gender 1=F 2=M	Age of initiating wear	OD UCVA	OS UCVA	OD Sphere Rx	OD CYL	OD AXIS	OD SE	OS SPHERE	OS CYL	OS AXIS	OS SE	OD FLATK	OD STEEPK	OD AVE K	OD DIFF	OS FLAT	OS STEEPK	OS AVE	OS DIFF	OD CCT	OS CCT	OD AL B	OS AL B	OD WTW	OS WTW	TOTAL WEARING MONTHS	OD LAST	OS LAST	OD PUPIL DIAM	OS PUPIL DIAM	OD EVAL	OS EVALUE	OD IOP	OS IOP	OD AL D	OD 12 months	OS AL DIFF	OS 12 MONTHS
1	14	0.08	0.1	-4			-4	-4			-4	43	43.75	43.375	0.75	43	44	43.5	1	537	530	25.36	25.36	11.3	11.4	38	25.57	25.46	3.86	4.05	0.63	0.5	14.7	13.2	0.0055	0.0663	0.1	0.0316
2	6	0.2	0.2	-3			-3	-2.75			-2.75	43.5	44.25	43.875	0.75	43.75	44.5	44.125	0.75	523	532	23.98	23.88	11.2	11.3	41	25.25	25.52	3.19	2.89	0.65	0.65	13.9	13.6	0.031	0.3717	1.64	0.48
2	8	0.05	0.05	-4			-4	-4	-0.5	180	-4.25	43.75	45	44.375	1.25	43.75	45.75	44.75	2	635	632	24.29	24.23	11.5	11.4	25	25.11	24.68	3.23	4.07	0.69	0.66	17.3	20	0.0328	0.3936	0.45	0.216
1	8	0.15	0.15	-2.25			-2.25	-2.25			-2.25	44.25	45.25	44.75	1	44.25	45	44.625	0.75	533	539	23.4	23.63	11.8	11.7	29	24.25	24.17	4.02	4.44	0.44	0.49	13.1	19.3	0.0293	0.3517	0.54	0.2234
2	14	0.2-	0.1	-2.25	-0.75	180	-2.625	-3.5	-0.5	180	-3.75	41.75	42.5	42.125	0.75	41.75	42.5	42.125	0.75	590	591	25.57	26.16	11.8	11.7	37	25.6	25.93	4.41	4.72	0.52	0.52	16	16	0.0008	0.0097	-0.23	-0.075
2	10	0.02	0.02	-4.5	-0.25	100	-4.625	-4.75			-4.75	42	42.25	42.125	0.25	41.75	42.25	42	0.5	534	543	25.55	25.62	12	12	30	25.55	25.58	5.02	4.9	0.55	0.57	15	16.5	0	0	-0.04	-0.016
1	9	0.05	0.05	-4			-4	-4			-4	43	44	43.5	1	42.75	44	43.375	1.25	572	571	24.76	24.96	11.8	11.9	19	24.89	25.12	4.54	4.22	0.6	0.63	14.9	17.9	0.0068	0.0821	0.16	0.1011
1	9	0.3	0.3	-2.25	-0.75	170	-2.625	-2.75	-0.5	170	-3	45	46.5	45.75	1.5	44.75	46	45.375	1.25	535	552	23.27	23.51	11.2	11.2	36	24.13	23.97	4.49	4.7	0.6	0.59	22	20	0.0239	0.2867	0.46	0.1533
2	9	0.15	0.15	-2.5			-2.5	-2.75			-2.75	41.25	42	41.625	0.75	41.5	42.25	41.875	0.75	514	495	25.62	25.72	12	12	42	26.13	26.29	3.68	3.6	0.54	0.39	13.1	16.5	0.0121	0.1457	0.57	0.1629
2	10	0.2+	0.2	-1.75			-1.75	-2			-2	42.5	43.25	42.875	0.75	42.25	43	42.625	0.75	538	540	24.67	24.8	11.5	11.5	27	24.88	25.03	4.2	4.02	0.55	0.57	11.1	12	0.0078	0.0933	0.23	0.1022
2	11	0.3+	0.3+	-1.75			-1.75	-1.25			-1.25	41.75	42.75	42.25	1	41.75	43.5	42.625	1.75	603	597	24.83	24.44	12	12	36	25.2	24.78	4.35	4.91	0.58	0.61	20	17	0.0103	0.1233	0.34	0.1133
1	10	0.1	0.1	-3.25	-0.75	180	-3.625	-3.5	-1.25	175	-4.125	43	44.5	43.75	1.5	42.75	44.75	43.75	2	555	538	24.86	24.74	11.6	11.5	28	25.88	25.45	3.01	3.05	0.72	0.65	23.6	20.9	0.0364	0.4371	0.71	0.3043
1	10	0.2	0.2-	-1.75			-1.75	-2			-2	42.5	43.5	43	1	42.5	43.75	43.125	1.25	529	531	24.55	24.5	11.3	11.4	29	25.28	25.17	4.45	3.43	0.66	0.63	15.7	20.1	0.0252	0.3021	0.67	0.2772
2	7	0.3	0.3	-1.25			-1.25	-1.25			-1.25	43.25	43.75	43.5	0.5	43.25	44	43.625	0.75	529	536	23.55	23.63	11.8	11.7	34	24.79	24.76	3.56	4.19	0.57	0.63	16.2	15.2	0.0365	0.4376	1.13	0.3988
2	12	0.15	0.2	-2.25			-2.25	-1.25	-1	170	-1.75	43.25	44.25	43.75	1	43.25	44.25	43.75	1	549	534	24.18	23.96	11.5	11.5	42	24.46	24.28	4.17	4.83	0.53	0.55	15.8	17.9	0.0067	0.08	0.32	0.0914
2	10	0.05	0.05	-3.75			-3.75	-3.5			-3.5	42.25	43	42.625	0.75	42.25	43	42.625	0.75	561	574	25.35	25.41	11.8	11.9	41	26	26.05	4.68	4.14	0.47	0.42	21	20	0.0159	0.1902	0.64	0.1873
2	12	0.05	0.05	-4.5	-0.5	30	-4.75	-3.25	-1	160	-3.75	44	45	44.5	1	43.5	45.25	44.375	1.75	555	552	25.2	24.85	11	11	37	25.52	25.39	4.39	4.66	0.65	0.77	17.6	18.9	0.0086	0.1038	0.54	0.1751
1	12	0.2-	0.2-	-1.5	-0.5	175	-1.75	-1.5	-0.5	180	-1.75	43.5	44.75	44.125	1.25	43.5	44.5	44	1	552	555	24	24.02	11.4	11.4	23	24.46	24.51	3.36	3.54	0.7	0.67	16.5	0.67	0.02	0.24	0.49	0.2557
2	7	0.2	0.2+	-2.25			-2.25	-2			-2	42.75	44	43.375	1.25	42.75	43.75	43.25	1	605	606	24.17	24.26	11.9	11.9	35	24.55	24.6	4.55	4.41	0.61	0.52	15.9	18	0.0109	0.1303	0.34	0.1166
1	12	0.05	0.15	-2			-2	-1	-0.75	180	-1.375	42	43.5	42.75	1.5	41.75	43.5	42.625	1.75	579	594	24.16	23.93	12	12	42	24.47	24.2	3.97	3.9	0.58	0.63	17.4	14.6	0.0074	0.0886	0.27	0.0771
1	9	0.3	0.4	-2.25			-2.25	-2			-2	44	44.75	44.375	0.75	44	44.75	44.375	0.75	581	593	23.5	24.42	11.4	11.4	41	24.07	24.07	3.39	3.92	0.63	0.61	16.1	15.7	0.0139	0.1668	-0.35	-0.102
2	9	0.05	0.05	-4.5			-4.5	-4.75			-4.75	42.25	43.25	42.75	1	42.25	43.25	42.75	1	572	565	25.24	25.36	11.8	11.7	36	26.26	26.53	3.69	4.52	0.42	0.41	18.9	20	0.0283	0.34	1.17	0.39
2	9	0.15	0.15	-2.5			-2.5	-2.75			-2.75	43.25	44.25	43.75	1	43.25	44	43.625	0.75	566	550	24.06	24.13	11.4	11.6	42	24.34	24.38	4.33	4.31	0.74	0.74	13.8	14.2	0.0067	0.08	0.25	0.0714
2	14	0.15	0.1	-1.5	-0.5	175	-1.75	-2	-0.5	180	-2.25	43	44.5	43.75	1.5	43	44.25	43.625	1.25	602	600	24.4	24.71	12	11.9	18	24.57	24.86	3.46	3.51	0.53	0.48	17.9	17.3	0.0094	0.1133	0.15	0.1
1	7	0.15	0.1	-3.5			-3.5	-3.75			-3.75	43.25	44	43.625	0.75	43.5	44.25	43.875	0.75	502	499	24.12	24.11	11.9	11.8	41	24.69	24.96	3.6	3.03	0.69	0.65	16.2	15.4	0.0139	0.1668	0.85	0.2488
2	9	0.2	0.2	-1.5			-1.5	-1.5			-1.5	42.25	43.5	42.875	1.25	42.25	43.5	42.875	1.25	578	574	23.88	23.9	11.7	11.8	29	24.4	24.62	4.02	3.53	0.59	0.63	14	16.6	0.0179	0.2152	0.72	0.2979
1	10	0.1-	0.1	-3.75	-0.5	180	-4	-4	-0.5	180	-4.25	42.5	43.5	43	1	42.25	43.75	43	1.5	561	572	24.41	24.42	11.7	11.7	22	24.39	24.43	4.64	3.67	0.73	0.74	13.6	19	-0.001	-0.011	0.01	0.0055
1	9	0.05-	0.05-	-4.5	-1	180	-5	-5	-0.5	155	-5.25	42.75	44.5	43.625	1.75	42.75	44.25	43.5	1.5	514	509	25.16	25.24	11.4	11.4	42	26.45	26.56	3.86	4.22	0.73	0.73	19.1	16.3	0.0307	0.3686	1.32	0.3771
1	11	0.2	0.4	-3.5	-0.75	20	-3.875	-2.25	-1.5	165	-3	44.5	45.75	45.125	1.25	44	46	45	2	530	524	24.12	23.88	11.4	11.9	38	24.13	24.1	3.47	2.87	0.51	0.58	15.5	18.3	0.0003	0.0032	0.22	0.0695
2	8	0.1	0.1	-2.5			-2.5	-2.5			-2.5	42.25	43	42.625	0.75	42.25	43	42.625	0.75	549	547	23.97	24.24	12.1	12.1	32	24.91	25.12	3.84	3.96	0.57	0.56	18.2	20	0.0294	0.3525	0.88	0.33
2	9	0.2	0.2	-3.5			-3.5	-3.5			-3.5	41.5	42.5	42	1	40.75	42	41.375	1.25	505	498	25.6	25.91	11.5	11.5	26	25.62	25.95	4.54	4.87	0.82	0.71	17	16.5	0.0008	0.0092	0.04	0.0185
1	8	0.3	0.3	-2.5	-0.5	90	-2.75	-2.5	-0.5	90	-2.75	44.25	44.5	44.375	0.25	44.5	44.75	44.625	0.25	635	643	23.45	23.43	11.4	11.3	31	24.41	24.35	4.91	3.12	0.45	0.29	18.9	20.5	0.031	0.3716	0.92	0.3561
1	10	0.4+	0.4	-2.5			-2.5	-2.75			-2.75	42.5	43	42.75	0.5	42.75	43.5	43.125	0.75	521	527	24.35	24.33	11.3	11.4	31	24.49	24.85	4.49	4.35	0.34	0.44	18.9	18	0.0045	0.0542	0.52	0.2013
1	9	0.2	0.2	-1.75	-1	165	-2.25	-2.5	-1.25	170	-3.125	41.75	43.5	42.625	1.75	41.75	43.5	42.625	1.75	572	580	24	24.12	12	12	32	24.42	24.65	5.02	3.5	0.65	0.53	17.2	16.6	0.0131	0.1575	0.53	0.1987
2	12	0.4	0.4	-1.25			-1.25	-1.25	-0.5	170	-1.5	42.25	43.25	42.75	1	42.25	43.5	42.875	1.25	565	570	24.26	24.17	11.5	11.5	38	25.1	24.93	5.15	4.6	0.74	0.58	15.5	15.9	0.0221	0.2653	0.76	0.24
1	8	0.3	0.3	-2			-2	-2			-2	43.5	43.75	43.625	0.25	43	43.75	43.375	0.75	569	563	24.28	24.3	11.9	11.9	37	25.41	25.03	4.06	4.44	0.55	0.49	14.1	17.1	0.0305	0.3665	0.73	0.2368
2	15	0.15	0.1	-3.5			-3.5	-3.5	-0.5	10	-3.75	42.5	43.25	42.875	0.75	42.25	43.5	42.875	1.25	564	560	25.76	25.82	11.8	11.8	39	25.82	25.81	3.27	3.78	0.57	0.61	13.5	14.5	0.0015	0.0185	-0.01	-0.003
1	10	0.1	0.1	-3.25	-0.5	180	-3.5	-3.25	-0.5	180	-3.5	42	44	43	2	41.75	43.5	42.625	1.75	574	569	24.31	24.33	11.3	11.3	27	24.39	24.44	3.82	3.27	0.57	0.52	11	12	0.003	0.0356	0.11	0.0489
1	9	0.6	0.5	-0.75			-0.75	-0.75			-0.75	42	43.25	42.625	1.25	42.25	43.5	42.875	1.25	528	515	23.78	23.78	11.6	11.5	36	25.36	25.12	3.84	4.48	0.68	0.69	16.3	16.6	0.0439	0.5267	1.34	0.4467
1	8	0.15	0.15	-2.25	-0.25	160	-2.375	-2.25	-0.5	10	-2.5	44.75	45.75	45.25	1	44.75	45.75	45.25	1	518	517	23.65	23.62	11.7	11.7	33	24.21	24.43	3.61	3.32	0.58	0.58	16.5	14.9	0.017	0.2036	0.81	0.2945
1	9	0.02	0.05	-4.75			-4.75	-4			-4	42.5	43.75	43.125	1.25	42.5	44	43.25	1.5	537	540	25.08	24.81	11.7	11.7	26	25.66	25.67	4.11	3.38	0.52	0.5	14.9	15.3	0.0223	0.2677	0.86	0.3969
1	14	0.05	0.2	-3			-3	-1.5			-1.5	42.25	43.25	42.75	1	42	43.5	42.75	1.5	575	571	25.33	24.61	11.7	11.7	35	25.53	25.1	3.41	3.26	0.56	0.63	20.6	20.6	0.0057	0.0686	0.49	0.168
2	11	0.1	0.1	-3.75	-0.75	175	-4.125	-3.75	-0.75	180	-4.125	44	45.5	44.75	1.5	44	45.5	44.75	1.5	556	548	24.36	24.39	11.4	11.3	27	25.26	25.13	3.77	3.73	0.62	0.59	12.9	12.6	0.0333	0.4	0.74	0.3289
2	8	0.2	0.2	-1.75	-0.5	160	-2	-1.75	-0.5	170	-2	41.25	42.75	42	1.5	41	42.75	41.875	1.75	554	549	24.36	24.42	11.2	11.2	37	25.2	25.23	3.23	3.28	0.86	0.83	20.6	18	0.0227	0.2724	0.81	0.2627
2	10	0.3+	0.15+	-1.25	-0.5	180	-1.5	-1.5	-1	180	-2	43	44.25	43.625	1.25	42.5	44	43.25	1.5	586	592	24.55	24.85	11.7	11.8	32	25.05	25.75	4.88	4.04	0.64	0.61	10.9	13.2	0.0156	0.1875	0.9	0.3375
2	10	0.05	0.2-	-2.75	-0.75	175	-3.125	-2	-0.75	175	-2.375	41.5	42.5	42	1	41.75	43	42.375	1.25	567	559	25.31	24.91	11.7	11.7	35	26.21	25.81	4.57	5.56	0.63	0.65	17.7	16.3	0.0257	0.3086	0.9	0.3086
2	10	0.2	0.2	-2.75			-2.75	-2.5			-2.5	43.5	44.25	43.875	0.75	43	43.75	43.375	0.75	606	603	23.88	24.02	11.7	11.7	32	24.21	24.23	3.88	3.4	0.57	0.63	16.1	17.1	0.0103	0.1238	0.21	0.0788
2	11	0.4-	0.5-	-2.5	-0.75	175	-2.875	-1.5	-1.25	170	-2.125	43.25	44.75	44	1.5	43.5	45	44.25	1.5	593	584	24.63	24.19	12.1	12.1	27	24.92	24.64	4.11	3.56	0.59	0.57	18.2	21.6	0.0107	0.1289	0.45	0.2
1	7	0.3-	0.3-	-2.25			-2.25	-1.75	-0.5	60	-2	42.25	43.5	42.875	1.25	42.5	43.5	43	1	542	528	24.48	24.22	11.7	11.6	36	25.16	25.1	4.66	3.59	0.59	0.6	10	9.4	0.0189	0.2267	0.88	0.2933
1	8	0.05	0.05	-3.25	-1.25	180	-3.875	-3.25	-1.25	180	-3.875	43.75	45.25	44.5	1.5	43.75	45.25	44.5	1.5	464	460	23.98	24.08	11.8	11.9	38	24.51	24.31	3.82	3.95	0.59	0.61	12.1	12.7	0.0139	0.1674	0.23	0.0726
2	8	0.6	0.5	-1.5			-1.5	-1.75	-0.5	180	-2	41.5	42.5	42	1	41.75	43	42.375	1.25	489	497	24.55	24.56	11.9	11.9	36	25.35	25.17	4.04	4.44	0.72	0.81	22.9	11.6	0.0222	0.2667	0.61	0.2033
2	7	0.05	0.05	-3.75			-3.75	-3.5			-3.5	43.25	44.5	43.875	1.25	43.25	44.75	44	1.5	571	576	24.77	24.65	10.6	10.6	37	25.43	25.33	3.69	3.81	0.51	0.6	12.8	14.7	0.0178	0.2141	0.68	0.2205
2	7	0.05	0.05	-4			-4	-4	-0.25	160	-4.125	44.25	45	44.625	0.75	44.25	45.25	44.75	1	596	581	24.61	24.55	11.5	11.6	21	24.94	24.88	3.87	3.73	0.57	0.48	12.4	12.4	0.0157	0.1886	0.33	0.1886
2	13	0.1	0.15-	-5.25			-5.25	-5			-5	42.75	43.5	43.125	0.75	42.25	43	42.625	0.75	561	537	26.25	26.44	12.2	12.3	37	26.38	26.56	4.38	5	0.47	0.46	12.6	12.5	0.0035	0.0422	0.12	0.0389
2	8	0.3	0.2	-1.5			-1.5	-1.5			-1.5	42.25	43.5	42.875	1.25	42.25	43.25	42.75	1	594	581	24.38	24.54	11.8	11.8	26	25.3	25.51	4.3	3.79	0.52	0.46	14.4	15.8	0.0354	0.4246	0.97	0.4477
1	10	0.04	0.05-	-3.75			-3.75	-3.75	-0.5	170	-4	42	43	42.5	1	42	43	42.5	1	609	608	25.09	25.21	11.7	11.7	34	25.69	25.62	3.71	3.27	0.45	0.53	17.5	17.9	0.0176	0.2118	0.41	0.1447
2	10	0.1	0.1	-3.75	-1.25	175	-4.375	-3.75	-1.25	175	-4.375	43	44.5	43.75	1.5	43.25	45	44.125	1.75	552	539	25.05	24.9	11.8	11.7	42	25.9	25.87	4.35	4.44	0.64	0.62	10.5	11	0.0202	0.2429	0.97	0.2771
2	7	0.3	0.5	-1.75			-1.75	-1			-1	43.75	44.25	44	0.5	43.5	44.25	43.875	0.75	543	553	24.12	23.85	11.5	11.5	35	25.03	24.87	4.65	4.49	0.28	0.5	15.1	13.1	0.026	0.312	1.02	0.3497
2	8	0.5+	0.5+	-1.5			-1.5	-1.25			-1.25	40.5	41.25	40.875	0.75	39.75	40.5	40.125	0.75	587	577	24.67	24.9	11.8	11.7	33	25.39	25.8	4.26	3.9	0.72	0.77	10.8	10.4	0.0218	0.2618	0.9	0.3273
2	9	0.3	0.1	-1.5	-0.75	180	-1.875	-2.75	-0.5	175	-3	42	43.5	42.75	1.5	42	43.25	42.625	1.25	509	496	25.07	25.62	12.3	12.2	36	26.05	25.96	3.1	3.09	0.56	0.63	10.7	10.7	0.0272	0.3267	0.34	0.1133
1	7	0.4	0.5+	-1.5			-1.5	-1.25			-1.25	42.25	43.25	42.75	1	42.25	43.5	42.875	1.25	521	524	24.03	23.8	11.4	11.3	24	24.95	24.71	3.96	4.54	0.7	0.68	12	12.2	0.0383	0.46	0.91	0.455

1	11	0.1	0.1	-1.75	-0.5	80	-2	-2.25			-2.25	42	42.5	42.25	0.5	41.5	42.25	41.875	0.75	497	503	24.91	25.11	12	12	38	25.27	25.28	3.38	3.32	0.58	0.63	14.5	16.4	0.0095	0.1137	0.17	0.0537
1	8	0.15	0.15	-4	-0.5	5	-4.25	-4	-0.5	10	-4.25	43	44.25	43.625	1.25	43.25	44.5	43.875	1.25	517	510	24.2	24.18	11.6	11.7	36	25.3	25.43	4.5	4.17	0.72	0.7	19.9	15.9	0.0306	0.3667	1.25	0.4167
1	12	0.3	0.15	-1.75			-1.75	-2.5			-2.5	41.25	41.75	41.5	0.5	41.75	42.25	42	0.5	517	532	24.47	24.98	12	11.9	37	25.35	24.71	3.39	4.22	0.44	0.63	16.6	17.8	0.0238	0.2854	-0.27	-0.088
1	12	0.2	0.15	-3.25			-3.25	-3.5			-3.5	42.5	43	42.75	0.5	42.5	43.25	42.875	0.75	533	522	25.19	25.15	11.9	11.9	35	24.93	24.86	3.16	3.24	0.59	0.59	18	19	-0.007	-0.089	-0.29	-0.099
1	10	0.4-	0.4-	-3.25			-3.25	-3.25			-3.25	43	43.25	43.125	0.25	42.75	43.25	43	0.5	538	539	24.97	24.98	11.4	11.5	36	25.57	25.67	4.08	4.34	0.65	0.71	18.7	16.1	0.0167	0.2	0.69	0.23
1	10	0.15	0.2	-3	-0.5	170	-3.25	-1.5	-0.75	180	-1.875	44	45.25	44.625	1.25	43.75	45.5	44.625	1.75	523	515	24.13	23.67	11.1	11.1	36	25.09	24.16	4.16	4.53	0.55	0.59	13.3	13.2	0.0267	0.32	0.49	0.1633
2	8	0.15-	0.15	-3.5	-0.5	175	-3.75	-3.25	-0.75	180	-3.625	44.5	46.25	45.375	1.75	44.25	46	45.125	1.75	535	536	23.79	23.74	11.5	11.4	42	23.99	24.12	3.25	3.56	0.66	0.71	10.8	11.7	0.0048	0.0571	0.38	0.1086
1	10	0.4	0.4	-2			-2	-2.25			-2.25	43.5	44.25	43.875	0.75	43.5	44.5	44	1	567	561	23.49	23.51	11.8	11.7	36	23.85	23.94	3.8	3.75	0.53	0.49	14	13.8	0.01	0.12	0.43	0.1433
1	12	0.05+	0.05+	-3.5	-0.75	145	-3.875	-3.5	-0.5	175	-3.75	41.5	42.75	42.125	1.25	41.5	43	42.25	1.5	515	517	25.61	25.33	11.6	11.6	31	25.73	25.61	3.24	3.67	0.66	0.66	14.4	12.7	0.0039	0.0465	0.28	0.1084
2	12	0.2+	0.1+	-2	-0.5	180	-2.25	-3	-1	180	-3.5	41.75	42.75	42.25	1	41.5	43	42.25	1.5	560	573	24.47	25.11	11.4	11.3	21	24.7	25.3	4.06	4.18	0.6	0.57	11.8	13.1	0.011	0.1314	0.19	0.1086
2	8	0.3	0.4	-1.75			-1.75	-1.25			-1.25	41.5	42	41.75	0.5	41.25	42	41.625	0.75	559	551	24.62	24.53	12	12	30	25.45	25.06	3.77	3.74	0.52	0.49	16	14.4	0.0277	0.332	0.53	0.212
2	8	0.2	0.2	-2.75			-2.75	-2.75			-2.75	42.75	43.75	43.25	1	43	44	43.5	1	537	544	24.7	24.05	12	12	36	25.73	25.81	3.01	2.92	0.66	0.58	13.6	10.7	0.0286	0.3433	1.76	0.5867
1	8	0.05	0.05	-4.25	-0.5	175	-4.5	-4	-1.25	180	-4.625	41.75	43.25	42.5	1.5	42	43.75	42.875	1.75	510	490	24.89	24.83	12.1	12.2	11	25.35	25.25	3.21	3.24	0.8	0.73	12.3	13.1	0.0418	0.5018	0.42	0.4582
1	8	0.3	0.15	-1.25	-0.75	175	-1.625	-2.25	-0.5	170	-2.5	40.5	42.25	41.375	1.75	40.75	42	41.375	1.25	593	586	25	25.5	11.7	11.7	35	25.73	26.32	3.56	4.17	0.74	0.66	16.9	19.2	0.0209	0.2503	0.82	0.2811
1	11	0.05	0.05	-3.25	-0.5	165	-3.5	-3.25	-0.5	180	-3.5	41.25	43.25	42.25	2	41.5	43.5	42.5	2	555	550	24.51	24.54	11.9	11.8	22	24.6	24.56	3.34	3.11	0.71	0.72	16.1	15.1	0.0041	0.0491	0.02	0.0109
1	13	0.15	0.2	-3.25			-3.25	-2.75	-0.75	170	-3.125	42.5	43.25	42.875	0.75	42.5	43.5	43	1	600	593	24.9	24.74	11.5	11.5	36	25.25	25.04	4.27	3.71	0.66	0.55	19.4	20.3	0.0097	0.1167	0.3	0.1
1	9	0.3	0.3	-1.75			-1.75	-1.75			-1.75	41.75	42.5	42.125	0.75	41.75	43.75	42.75	2	571	570	24.38	24.19	11.5	11.5	24	25.09	25.26	3.26	3.17	0.43	0.39	13.1	11.7	0.0296	0.355	1.07	0.535
2	10	0.15-	0.15	-2			-2	-1.75			-1.75	43.5	44.75	44.125	1.25	43.5	44.25	43.875	0.75	492	502	23.39	23.5	11	11.1	35	23.6	23.85	3.27	4.43	0.47	0.49	9.3	9.3	0.006	0.072	0.35	0.12
1	10	0.05	0.05	-4			-4	-4			-4	41.75	42.5	42.125	0.75	41.75	42.75	42.25	1	550	544	25.35	25.25	11.5	11.5	35	26.12	25.93	3.32	3.73	0.65	0.52	12.3	13.7	0.022	0.264	0.68	0.2331
1	11	0.05	0.05	-5			-5	-4.5			-4.5	42.25	42.75	42.5	0.5	42	42.75	42.375	0.75	575	573	25.9	25.74	11.7	11.7	36	26.19	25.96	3.77	3.75	0.26	0.1	18	16	0.0081	0.0967	0.22	0.0733
1	7	0.2	0.2	-2.25			-2.25	-2			-2	42.5	43.25	42.875	0.75	42.25	42.75	42.5	0.5	568	547	24.05	24.05	12	12	33	25.59	25.66	3.32	3.83	0.55	0.52	15	15	0.0467	0.56	1.61	0.5855
1	8	0.15	0.1+	-3.75			-3.75	-4.25			-4.25	43	44	43.5	1	43	44	43.5	1	524	532	24.72	24.76	11.8	11.7	27	25.39	25.47	4.59	4.55	0.61	0.67	11.4	9.5	0.0248	0.2978	0.71	0.3156
1	9	0.1	0.1	-2.5			-2.5	-3			-3	41.5	42.25	41.875	0.75	41.5	42.5	42	1	556	562	24.77	24.89	11.3	11.4	21	24.85	25.02	3.43	4.34	0.33	0.47	12.9	12	0.0038	0.0457	0.13	0.0743
2	8	0.05	0.05	-3.5			-3.5	-3.75			-3.75	42	43	42.5	1	42	43	42.5	1	494	497	25.34	25.45	11.6	11.6	35	25.84	26.1	4.14	4.01	0.36	0.64	13.7	12.4	0.0143	0.1714	0.65	0.2229
2	14	0.05	0.4	-3.5	-0.5	165	-3.75	-1.25	-0.5	20	-1.5	43.5	44.75	44.125	1.25	43.5	44.75	44.125	1.25	532	514	24.8	23.68	11.8	11.8	37	24.77	23.66	4.15	3.03	0.62	0.63	14.7	16.9	-0.001	-0.01	-0.02	-0.006
1	10	0.4+	0.5	-1.25	-0.5	160	-1.5	-1	-0.5	180	-1.25	43.25	45	44.125	1.75	43.25	45.25	44.25	2	551	561	23.92	23.73	11.9	11.8	23	24.4	24.43	4.07	4.05	0.72	0.73	15.7	14.8	0.0209	0.2504	0.7	0.3652
1	9	0.05-	0.05	-4	-0.75	165	-4.375	-4	-0.5	180	-4.25	42.25	43.75	43	1.5	42	43.5	42.75	1.5	562	560	25.45	25.46	12	12	31	26.23	26.41	4.06	4.11	0.69	0.63	14.5	15.2	0.0252	0.3019	0.95	0.3677
1	7	0.15	0.15	-3			-3	-3			-3	41.25	42.5	41.875	1.25	41.25	42	41.625	0.75	527	530	24.89	24.77	11.8	11.8	34	25.84	25.87	3.63	3.97	0.74	0.78	18.5	18.1	0.0279	0.3353	1.1	0.3882
1	14	0.15	0.2	-1.75	-0.75	170	-2.125	-1.25	-0.5	180	-1.5	42.25	44.25	43.25	2	42.25	44.75	43.5	2.5	551	545	24.72	24.45	12.1	12.1	31	25.09	24.91	3.68	3.83	0.64	0.67	15.7	13.9	0.0119	0.1432	0.46	0.1781
2	8	0.05	0.05	-4.5			-4.5	-4.25	-0.5	180	-4.5	42.5	42.5	42.5	0	42.25	42.5	42.375	0.25	547	548	25.63	25.68	11.8	11.8	29	26.48	26.33	3.72	3.98	0.52	0.39	16	16.6	0.0293	0.3517	0.65	0.269
2	9	0.1	0.1	-3.5	-0.5	90	-3.75	-3.25			-3.25	42	42.25	42.125	0.25	42	42.25	42.125	0.25	546	553	24.59	24.41	11.7	11.6	19	24.95	24.65	3.85	4.08	0.57	0.54	13.8	12.5	0.0189	0.2274	0.24	0.1516
2	11	0.4	0.5	-1			-1	-0.75	-0.5	170	-1	42.75	43.75	43.25	1	43	44.25	43.625	1.25	540	553	24.33	24.14	11.4	11.4	36	24.85	24.73	3.56	3.52	0.59	0.68	11.4	11.1	0.0144	0.1733	0.59	0.1967
1	9	0.1-	0.1-	-3.25	-0.75	165	-3.625	-3	-1	5	-3.5	45	46.75	45.875	1.75	45	46.5	45.75	1.5	515	511	23.56	23.47	11.3	11.3	28	24.08	24.18	3.04	4.04	0.66	0.66	15.5	14.6	0.0186	0.2229	0.71	0.3043
2	10	0.05	0.05	-4.5	-1	170	-5	-3.75	-1.5	180	-4.5	43	44	43.5	1	42.5	44	43.25	1.5	551	550	25	24.75	11.5	11.5	35	25.54	25.27	4.15	4.42	0.58	0.52	15.8	14.3	0.0154	0.1851	0.52	0.1783
2	11	0.1	0.1	-2.25	-0.5	170	-2.5	-2.25	-0.75	180	-2.625	41.75	43.25	42.5	1.5	41.75	43	42.375	1.25	548	548	25.59	25.54	11.7	11.7	36	26.6	26.01	4.36	4.5	0.63	0.58	17.2	15.7	0.0281	0.3367	0.47	0.1567
1	11	0.2	0.3	-2.25			-2.25	-2.25	-0.5	90	-2.5	44.5	45.25	44.875	0.75	44.25	44.75	44.5	0.5	516	522	23.22	23.36	11.3	11.4	31	24.3	24.45	3.15	3.55	0.54	0.56	13.1	14.7	0.0348	0.4181	1.09	0.4219
2	8	0.3	0.3	-1.75	-0.5	175	-2	-1.75			-1.75	43	44	43.5	1	43	44.5	43.75	1.5	559	574	23.93	23.88	11.4	11.4	32	24.88	24.71	4.13	4.55	0.59	0.61	15.9	0.61	0.0297	0.3563	0.83	0.3113
1	13	0.05	0.05	-3.75	-1	5	-4.25	-4	-1	175	-4.5	42.75	44	43.375	1.25	43	44.25	43.625	1.25	544	560	25.82	25.95	12.1	12	35	25.92	26.02	2.98	3.04	0.51	0.52	14	15.4	0.0029	0.0343	0.07	0.024
2	10	0.1	0.1	-2.5			-2.5	-2.75	-0.75	175	-3.125	43.75	44.5	44.125	0.75	43.75	44.25	44	0.5	563	555	24.44	24.44	11.6	11.6	26	24.77	24.97	3.26	3.68	0.55	0.53	13.1	12.5	0.0127	0.1523	0.53	0.2446
2	8	0.2	0.3	-3			-3	-3			-3	42	43	42.5	1	42	43	42.5	1	516	522	24.89	24.98	12.3	12.3	24	25.52	25.47	4.62	4.43	0.55	0.55	14.9	16.1	0.0263	0.315	0.49	0.245
2	8	0.15	0.1	-3	-0.75	180	-3.375	-3	-0.75	175	-3.375	43.25	44.75	44	1.5	43	44.75	43.875	1.75	539	526	24.03	23.95	11.8	11.9	35	24.97	24.59	4.01	3.67	0.59	0.55	16.8	15.9	0.0269	0.3223	0.64	0.2194
1	12	0.3	0.3	-1.5			-1.5	-1.75			-1.75	41.5	42	41.75	0.5	41.25	41.75	41.5	0.5	573	557	24.74	25.03	11.5	11.6	28	24.95	24.97	4.75	3.89	0.25	0.44	11.7	11.1	0.0075	0.09	-0.06	-0.026
1	12	0.05-	0.05-	-4.5			-4.5	-5.5			-5.5	44	45	44.5	1	44.25	45	44.625	0.75	547	558	24.97	25.21	11.2	11.3	35	24.97	25.21	3.34	4.07	0.63	0.63	21.9	22	0	0	0	0
1	8	0.2+	0.2-	-2			-2	-2	-0.5	170	-2.25	42.25	43	42.625	0.75	42.25	43.25	42.75	1	533	544	24.3	24.28	11.6	11.6	35	25.74	25.65	4.41	3.94	0.53	0.58	15.1	15.5	0.0411	0.4937	1.37	0.4697
1	9	0.3+	0.3	-1.75			-1.75	-1.75			-1.75	43.75	44.5	44.125	0.75	43.5	44.25	43.875	0.75	557	547	23.89	24.01	11.5	11.5	35	24.31	24.45	3.34	3.55	0.66	0.58	11.2	9.1	0.012	0.144	0.44	0.1509
2	8	0.1	0.15	-3.25			-3.25	-2.75	-0.5	175	-3	42.75	43.25	43	0.5	42.5	43.25	42.875	0.75	576	567	24.64	24.61	11.6	11.7	37	25.62	25.45	3.64	2.72	0.62	0.58	18.2	16.2	0.0265	0.3178	0.84	0.2724
1	9	0.05	0.05	-4.75			-4.75	-5.25	-1.25	180	-5.875	41	42.75	41.875	1.75	42.25	43	42.625	0.75	575	573	25.9	26.02	12	12	35	25.84	25.99	3.89	3.87	0.56	0.56	16.5	17	-0.002	-0.021	-0.03	-0.01
2	8	0.15	0.15	-2			-2	-1.75			-1.75	44	44.75	44.375	0.75	44.25	45	44.625	0.75	556	563	23.82	23.75	11.5	11.5	15	24.17	24.02	3.7	3.28	0.59	0.63	15.1	16.3	0.0233	0.28	0.27	0.216
2	8	0.05	0.05-	-4.25	-1	180	-4.75	-4.75	-1	180	-5.25	42.25	44.25	43.25	2	42.5	44.25	43.375	1.75	616	619	25.63	25.75	12.4	12.4	36	26.3	26.5	4.82	4.47	0.58	0.5	14.7	16.5	0.0186	0.2233	0.75	0.25
2	7	0.1+	0.1+	-3.25	-1	10	-3.75	-3.25	-1	170	-3.75	42.5	43.75	43.125	1.25	42.75	43.5	43.125	0.75	547	548	24.11	24.15	11.7	11.8	31	24.81	24.93	3.5	4.33	0.68	0.64	12.6	11.7	0.0226	0.271	0.78	0.3019
2	9	0.2	0.2-	-2.75			-2.75	-2.75			-2.75	42.5	43.5	43	1	42.25	43	42.625	0.75	524	534	24.6	24.84	11.3	11.3	29	25.84	25.63	3.73	3.68	0.59	0.6	10.8	11.9	0.0428	0.5131	0.79	0.3269
2	7	0.05	0.05	-4	-0.5	15	-4.25	-4	-0.5	170	-4.25	42	42.75	42.375	0.75	42	43	42.5	1	511	524	24.37	24.19	11.5	11.6	32	24.59	24.51	4.32	4.01	0.61	0.61	12.8	12.2	0.0069	0.0825	0.32	0.12
1	8	0.3	0.15	-1.75	-0.5	170	-2	-2.25	-0.5	10	-2.5	45	46.5	45.75	1.5	45.5	46.75	46.125	1.25	565	556	23.29	23.23	11.5	11.4	13	23.44	23.45	4.12	4.03	0.52	0.54	15.5	14.8	0.0115	0.1385	0.22	0.2031
1	7	0.3	0.4	-1.5			-1.5	-1			-1	42.25	43.25	42.75	1	42	43	42.5	1	635	624	24.32	24.5	12.2	12.2	40	25.18	25.42	3.83	5.48	0.61	0.59	15.8	15.9	0.0215	0.258	0.92	0.276
1	9	0.05	0.05	-3.75			-3.75	-3.75			-3.75	42	42.5	42.25	0.5	42	42.5	42.25	0.5	616	616	24.47	25.55	11.7	11.7	28	25.74	25.85	4.13	4.18	0.45	0.44	18.7	19.5	0.0454	0.5443	0.3	0.1286
1	12	0.1	0.1	-4	-0.75	170	-4.375	-3.25	-0.75	180	-3.625	41.5	43	42.25	1.5	41.5	43	42.25	1.5	605	610	26.06	25.7	11.8	11.8	24	26.4	25.97	3.15	3.47	0.46	0.48	17.3	17	0.0142	0.17	0.27	0.135
2	11	0.1	0.2	-2.5	-0.5	175	-2.75	-1.25	-1.25	175	-1.875	43.75	44.75	44.25	1	43.5	45	44.25	1.5	509	512	24.45	24.07	11.8	11.8	36	24.91	24.97	3.95	3.81	0.7	0.74	14.3	11.6	0.0128	0.1533	0.9	0.3
2	7	0.4-	0.4-	-1.5			-1.5	-1.5			-1.5	43.25	44.5	43.875	1.25	43	44.25	43.625	1.25	544	563	23.9	24.07	12.2	12.2	27	24.52	24.72	4.8	4.49	0.56	0.6	17.9	20.9	0.023	0.2756	0.65	0.2889
1	10	0.4-	0.2	-1.5	-0.5	175	-1.75	-2	-0.75	170	-2.375	40.75	42.25	41.5	1.5	41	42.25	41.625	1.25	541	539	25.34	25.52	11.8	11.8	40	26.41	26.44	3.84	4.06	0.6	0.65	15.7	14.9	0.0268	0.321	0.92	0.276
1	10	0.3	0.2	-1.25			-1.25	-1.5			-1.5	41.75	42.25	42	0.5	41.75	42.25	42	0.5	581	562	24.89	25.17	11.9	11.9	35	25.47	25.77	5.04	4.21	0.49	0.33	11.1	12.1	0.0166	0.1989	0.6	0.2057
1	9	0.2	0.4	-1.75	-0.5	180	-2	-0.75	-1	180	-1.25	44.25	45.25	44.75	1	43.5	45.25	44.375	1.75	576	564	23.52	23.26	11.3	11.3	35	24.26	24.1	4.16	3.77	0.4	0.41	16.5	15.8	0.0211	0.2537	0.84	0.288
2	13	0.05	0.05	-3			-3	-2.75	-0.5	155	-3	42.25	42.75	42.5	0.5	41.75	42.5	42.125	0.75	514	499	25.05	25.12	12.6	12.6	37	25.37	25.24	3.34	3.25	0.59	0.58	15.7	18.3	0.0086	0.1038	0.12	0.0389
1	11	0.1	0.1	-3			-3	-2.5	-0.75	175	-2.875	42.25	43.5	42.875	1.25	41.75	43.25	42.5	1.5	545	546	25.07	25.11	12.2	12.2	23	25.85	25.73	2.89	2.77	0.44	0.43	13.4	16.1	0.0339	0.407	0.62	0.3235
1	14	0.05	0.1	-2.75			-2.75	-2	-0.5	165	-2.25	43.75	44.75	44.25	1	43.75	44.75	44.25	1	516	511	24.78	24.7	12.2	12.2	29	24.88	24.87	4.08	3.76	0.41	0.37	16.7	17.7	0.0034	0.0414	0.17	0.0703

1	10	0.1	0.1	-2	-0.5	160	-2.25	-3.25			-3.25	41.5	42	41.75	0.5	41.5	42	41.75	0.5	565	557	24.58	25.17	12	12	31	25.08	25.96	3.97	3.18	0.33	0.34	17	16	0.0161	0.1935	0.79	0.3058
1	12	0.3+	0.6	-1.75	-0.75	170	-2.125	-1	-1	180	-1.5	42.25	44	43.125	1.75	42.25	43.75	43	1.5	575	579	24.56	24.39	11.7	11.6	32	24.86	24.7	3.3	3.32	0.54	0.57	20.5	19.1	0.0094	0.1125	0.31	0.1163
2	11	0.1	0.1	-2.5			-2.5	-2.5			-2.5	42	43	42.5	1	42.5	43.75	43.125	1.25	557	568	25.25	25.02	11.7	11.7	30	25.57	25.45	4.06	4.1	0.08	0.22	12.6	11	0.0107	0.128	0.43	0.172
1	10	0.02	0.02	-4	-0.5	180	-4.25	-3.5	-0.75	175	-3.875	42.25	43.5	42.875	1.25	42	43.5	42.75	1.5	536	539	26.37	26.35	11.8	11.8	33	26.78	26.74	4.06	3.54	0.46	0.39	14.4	13.3	0.0124	0.1491	0.39	0.1418
2	10	0.4	0.4	-1.75	-0.75	180	-2.125	-1.75	-0.75	180	-2.125	42.25	42.25	42.25	0	42.25	42.25	42.25	0	586	588	24.81	24.89	11.8	11.8	34	25.45	25.65	3.83	3.75	0.61	0.59	15	16	0.0188	0.2259	0.76	0.2682
2	11	0.15	0.15	-2.5			-2.5	-2.75			-2.75	43.25	43.75	43.5	0.5	43	43.5	43.25	0.5	558	558	25.16	25.43	11.8	11.8	27	25.25	25.6	4.37	5.03	0.48	0.48	17	19	0.0033	0.04	0.17	0.0756
1	11	0.05	0.05	-5			-5	-4.5	-0.5	170	-4.75	44.25	45.25	44.75	1	44.25	45.5	44.875	1.25	559	552	24.8	24.72	11.5	11.5	34	24.93	25.09	3.57	3.67	0.23	0.4	16.3	14.7	0.0038	0.0459	0.37	0.1306
1	14	0.05	0.1	-3.25	-0.5	10	-3.5	-2.75	-1	150	-3.25	44	45.25	44.625	1.25	44.25	45.5	44.875	1.25	558	552	24.03	23.98	11.3	11.3	30	24.41	24.41	3.45	3.22	0.61	0.26	13.4	16.9	0.0127	0.152	0.43	0.172
2	11	0.15	0.1	-2.25			-2.25	-2.5			-2.5	42.25	43.5	42.875	1.25	42.25	43.25	42.75	1	538	547	24.58	24.67	11.1	11.1	26	25.2	25.33	3.12	2.98	0.42	0.54	13.1	12.1	0.0238	0.2862	0.66	0.3046
2	8	0.1-	0.1-	-4			-4	-4			-4	43.5	44.52	44.01	1.02	43.75	44.75	44.25	1	522	527	25.17	25.19	11.7	11.8	35	26.3	26.39	3.79	4.12	0.64	0.63	16.7	20.6	0.0323	0.3874	1.2	0.4114
1	12	0.05	0.4	-3.75			-3.75	-1.25	-1	10	-1.75	44.25	45.5	44.875	1.25	44	46	45	2	500	503	24.25	23.42	11.4	11.5	18	24.45	24.04	3.61	3.01	0.55	0.56	12.6	12.4	0.0111	0.1333	0.62	0.4133
2	12	0.15	0.15	-3.75	-1	5	-4.25	-2.75	-1.25	175	-3.375	43.25	45.25	44.25	2	43.25	45.25	44.25	2	574	562	25.05	24.56	11.5	11.6	29	25.58	25.16	4.21	4.22	0.2	0.45	16.6	15	0.0183	0.2193	0.6	0.2483
1	8	0.05	0.05	-3.5			-3.5	-3.5			-3.5	42.5	43.25	42.875	0.75	42.75	43.75	43.25	1	533	535	24.65	24.52	12.1	12.1	19	24.88	24.75	3.96	3.66	0.57	0.56	17.8	18.3	0.0121	0.1453	0.23	0.1453
1	9	0.05	0.05	-3.25			-3.25	-3.25			-3.25	41.75	43	42.375	1.25	42.25	43.5	42.875	1.25	580	568	24.88	24.7	12	11.9	30	25.41	25.2	4.18	4.57	0.46	0.49	15	14.1	0.0177	0.212	0.5	0.2
2	10	0.15	0.15	-3.5			-3.5	-3.5	-0.5	160	-3.75	41.5	42.75	42.125	1.25	41.75	43.25	42.5	1.5	637	637	25.57	25.54	11.9	11.9	28	25.84	25.8	3.25	3.62	0.38	0.32	19.11	18.6	0.0096	0.1157	0.26	0.1114
1	8	0.04	0.05	-3.25	-0.5	175	-3.5	-3.5	-0.75	180	-3.875	43.25	44.5	43.875	1.25	43.5	44.75	44.125	1.25	587	573	24.51	24.66	11.9	11.9	35	25.24	25.34	4.64	4.4	0.43	0.49	17.2	15.7	0.0209	0.2503	0.68	0.2331
2	9	0.05	0.05	-3			-3	-2.75			-2.75	44.5	45.5	45	1	44.5	45.75	45.125	1.25	507	526	23.94	23.95	11.8	11.8	27	24.44	24.37	3.83	3.35	0.54	0.5	14.9	14.1	0.0185	0.2222	0.42	0.1867
1	10	0.15	0.15	-3.25			-3.25	-3			-3	42	42.25	42.125	0.25	42	42.25	42.125	0.25	571	587	25.05	24.87	11.9	11.9	27	25.02	24.86	3.51	3.67	0.44	0.46	16.4	19.6	-0.001	-0.013	-0.01	-0.004
1	11	0.2+	0.2+	-1.25			-1.25	-1.25			-1.25	44	44.75	44.375	0.75	44.25	45	44.625	0.75	540	539	23.6	23.47	11.2	11.3	23	24.24	24.14	3.78	3.65	0.55	0.59	10.6	12.4	0.0278	0.3339	0.67	0.3496
1	10	0.05	0.05-	-5.25	-0.25	15	-5.375	-5.5			-5.5	42.25	43.25	42.75	1	42.25	43	42.625	0.75	552	552	26.23	26.24	11.6	11.7	26	26.63	26.37	3.6	3.45	0.27	0.47	13.9	14.4	0.0154	0.1846	0.13	0.06
1	13	0.15	0.05	-3.75			-3.75	-4.5	-0.25	180	-4.625	41.5	42.5	42	1	41.75	43	42.375	1.25	544	549	25.54	25.75	11.9	12	35	25.35	25.38	3.22	3.58	0.46	0.54	15.1	19.8	-0.005	-0.065	-0.37	-0.127
1	9	0.05	0.05	-4			-4	-4.25			-4.25	41.5	42.25	41.875	0.75	41.75	42.75	42.25	1	585	576	25.29	25.35	11.6	11.6	39	26.59	26.58	4.71	4.65	0.6	0.56	13.3	14.4	0.0333	0.4	1.23	0.3785
1	10	0.15	0.3	-2.5			-2.5	-1.25	-0.5	5	-1.5	41	42	41.5	1	40.5	41.5	41	1	555	555	25.17	24.62	11.6	11.5	22	25.4	24.96	3.5	2.66	0.51	0.52	13.6	11.7	0.0105	0.1255	0.34	0.1855
1	15	0.1	0.05	-3.25	-1.25	170	-3.875	-4	-1	180	-4.5	43	44.25	43.625	1.25	42.75	44.25	43.5	1.5	559	556	25.32	25.59	11.9	11.9	32	25.58	25.73	2.7	2.6	0.41	0.45	11.3	12.4	0.0081	0.0975	0.14	0.0525
1	10	0.2-	0.2	-3.5			-3.5	-3.25			-3.25	42.5	43	42.75	0.5	42.75	43.5	43.125	0.75	545	554	25.62	25.04	11.7	11.7	28	25.46	25.33	4.06	3.57	0.42	0.52	15.2	14.2	-0.006	-0.069	0.29	0.1243
1	11	0.05	0.05	-4			-4	-3.75	-0.5	170	-4	41.5	42.5	42	1	41.25	42.25	41.75	1	549	540	25.43	25.39	11.7	11.7	22	25.66	25.49	4.8	4.07	0.52	0.56	15	15	0.0105	0.1255	0.1	0.0545
2	12	0.15-	0.15-	-3.25	-0.5	15	-3.5	-3	-0.5	165	-3.25	41.75	42.5	42.125	0.75	41.25	42.5	41.875	1.25	593	589	25.83	26.01	11.7	11.7	33	26.42	26.56	4.03	5.34	0.43	0.34	16	16	0.0179	0.2145	0.55	0.2
1	11	0.05	0.05	-3.5	-1	180	-4	-3.75	-1	170	-4.25	43.5	44.75	44.125	1.25	43.75	45	44.375	1.25	614	600	24.77	24.76	11.1	11.1	28	25.42	25.23	2.95	3.15	0.61	0.7	16.6	17.5	0.0232	0.2786	0.47	0.2014
1	10	0.15	0.15+	-2.25			-2.25	-2			-2	42	42.75	42.375	0.75	42	42.5	42.25	0.5	561	569	24.59	24.43	11.4	11.4	35	25.48	25.46	3.35	3.84	0.18	0.46	17.1	17.9	0.0254	0.3051	1.03	0.3531
1	10	0.05-	0.05-	-5.5	-0.5	180	-5.75	-5	-1	165	-5.5	42.75	44.25	43.5	1.5	42.5	44	43.25	1.5	541	547	25.62	25.55	11.7	11.7	35	25.64	25.54	3.85	4.35	0.45	0.48	18.3	16	0.0006	0.0069	-0.01	-0.003
1	9	0.05	0.05	-3			-3	-3.75			-3.75	45.5	46	45.75	0.5	45.5	46.25	45.875	0.75	529	526	23.54	23.84	11	11	33	24.01	24.55	3.66	4.75	0.41	0.61	16.2	14	0.0142	0.1709	0.71	0.2582
1	10	0.05	0.05	-4.75			-4.75	-5	-0.5	90	-5.25	43.25	44	43.625	0.75	43.5	44	43.75	0.5	538	544	24.72	24.83	11.4	11.4	26	25.43	25.65	2.75	3.63	0.47	0.51	12.9	12.8	0.0273	0.3277	0.82	0.3785
2	8	0.1	0.1	-3.5			-3.5	-3.75			-3.75	42.75	42.75	42.75	0	42.75	42.75	42.75	0	579	569	25.03	25.32	12	12	20	25.02	25.24	4.26	3.59	0.35	0.32	18.1	15.7	-0.001	-0.006	-0.08	-0.048
1	9	0.2	0.2	-2.25			-2.25	-2.25			-2.25	43.25	44.25	43.75	1	43.25	44.5	43.875	1.25	532	532	24.32	24.3	11.4	11.4	36	24.57	24.48	4.67	4.71	0.52	0.56	18.2	15.8	0.0069	0.0833	0.18	0.06
2	7	0.4	0.4	-1.5			-1.5	-1.25			-1.25	44.75	46	45.375	1.25	44.25	46	45.125	1.75	548	543	22.73	22.75	11.2	11.2	24	23.25	23.4	3.73	3.82	0.56	0.49	17.5	14.8	0.0217	0.26	0.65	0.325
1	8	0.4	0.3	-1.25			-1.25	-1.25			-1.25	41.5	41.75	41.625	0.25	41.25	41.5	41.375	0.25	534	527	24.14	24.26	11.9	12	22	24.96	25.19	4.05	3.49	0.34	0.49	13.7	11.8	0.0373	0.4473	0.93	0.5073
1	13	0.15	0.2	-2.25			-2.25	-1.75	-0.5	145	-2	43.5	44.75	44.125	1.25	43.75	45.25	44.5	1.5	599	609	24.41	24.1	11.2	11.2	32	24.64	24.47	4.26	4.35	0.39	0.72	18.9	18.9	0.0072	0.0863	0.37	0.1387
2	6	0.15	0.2	-2	-0.5	15	-2.25	-1.75	-0.5	165	-2	43.75	45	44.375	1.25	43.5	44.75	44.125	1.25	474	469	23.59	23.67	12.1	12.1	33	25.09	24.94	3.78	3.74	0.48	0.49	9.6	8.7	0.0455	0.5455	1.27	0.4618
2	14	0.2	0.2+	-3			-3	-2.75			-2.75	42.75	43.25	43	0.5	42.25	43	42.625	0.75	604	604	25.08	24.28	12	11.5	38	25.07	25.03	2.99	2.72	0.65	0.63	12.6	14.4	-3E-04	-0.003	0.75	0.2368
2	7	0.05	0.05	-3.25			-3.25	-3.25			-3.25	42.5	43.5	43	1	42.25	43	42.625	0.75	576	569	24.91	25.14	12.1	12.1	37	25.29	25.34	3.53	3.15	0.53	0.52	17.1	16.3	0.0103	0.1232	0.2	0.0649
1	10	0.1	0.1	-4.5	-1	170	-5	-4.5	-1.5	180	-5.25	42.5	44	43.25	1.5	42.25	44.5	43.375	2.25	565	551	25.09	25.12	11.5	11.4	36	25.17	24.95	4.92	4.63	0.52	0.39	16.4	15.3	0.0022	0.0267	-0.17	-0.057
1	10	0.05	0.05	-4.5	-1	180	-5	-4.25	-1	175	-4.75	43.25	44.75	44	1.5	43.75	45.25	44.5	1.5	522	526	24.72	24.5	10.7	10.8	33	25.01	24.8	3.7	3.39	0.6	0.64	13.7	12.9	0.0088	0.1055	0.3	0.1091
2	8	0.5	0.4	-0.75			-0.75	-1			-1	42.5	43.5	43	1	42	43	42.5	1	547	554	24.47	24.64	12.2	12.3	33	25.08	25.14	4.49	3.48	0.6	0.56	15.8	17.9	0.0185	0.2218	0.5	0.1818
1	9	0.2	0.3	-2.25			-2.25	-2			-2	44.75	46.25	45.5	1.5	45.25	46.5	45.875	1.25	551	548	23.31	23.07	12	11.9	29	24.01	23.49	3.9	4.7	0.55	0.62	14.4	14.1	0.0241	0.2897	0.42	0.1738
2	10	0.05	0.05	-4	-0.5	180	-4.25	-3.75	-0.5	180	-4	43.25	44.25	43.75	1	43	43.75	43.375	0.75	563	551	24.76	24.85	11.8	11.9	13	25.03	25.12	3.81	3.74	0.36	0.41	18	16.8	0.0208	0.2492	0.27	0.2492
2	8	0.1	0.1	-4	-0.5	5	-4.25	-4	-0.5	170	-4.25	43	44.25	43.625	1.25	43	44.25	43.625	1.25	531	548	24.96	24.85	11.7	11.7	31	25.55	25.15	4.02	4.11	0.24	0.32	14.3	17.9	0.019	0.2284	0.3	0.1161
2	14	0.15	0.2	-3			-3	-2.75			-2.75	43.25	44.25	43.75	1	43.5	44.75	44.125	1.25	616	612	24.46	24.39	12	11.9	18	24.39	24.3	3.38	3.57	0.6	0.59	17.5	17.6	-0.004	-0.047	-0.09	-0.06
1	14	0.05	0.1	-4.5	-0.75	180	-4.875	-3.75	-0.5	175	-4	44.25	45.5	44.875	1.25	44	45.5	44.75	1.5	576	583	24.7	24.3	11.4	11.4	32	24.32	24.03	4.08	3.87	0.26	0.42	20	17	-0.012	-0.143	-0.27	-0.101
2	11	0.05-	0.05	-4			-4	-3.75			-3.75	42.25	43.75	43	1.5	42.25	43.5	42.875	1.25	580	571	26.11	26.12	11.8	11.8	29	26.24	26.23	4.7	4.73	0.48	0.43	13.7	13.3	0.0045	0.0538	0.11	0.0455
1	9	0.15	0.5+	-1.75			-1.75	-1	-0.5	165	-1.25	41.75	42.75	42.25	1	41.75	43	42.375	1.25	605	620	24.15	23.72	11.8	11.7	25	25.05	24.43	3.79	4.28	0.6	0.73	15.9	13.5	0.036	0.432	0.71	0.3408
1	10	0.15	0.15	-3	-0.75	165	-3.375	-2.75	-0.75	5	-3.125	42.5	44	43.25	1.5	42.5	43.75	43.125	1.25	518	517	24.67	24.62	12.1	12.1	16	24.85	24.78	4.43	3.9	0.56	0.57	19.4	16.1	0.0113	0.135	0.16	0.12
1	8	0.3	0.4	-1.25			-1.25	-1.25			-1.25	43	43.25	43.125	0.25	42.75	43.25	43	0.5	530	539	23.66	23.65	11.5	11.4	27	24.22	24.28	3.28	3.67	0.46	0.54	18.5	18.6	0.0207	0.2489	0.63	0.28
2	9	0.15	0.15	-1.75			-1.75	-2	-0.5	170	-2.25	41.5	42.5	42	1	41	42.25	41.625	1.25	566	564	25.05	25.32	12	12	28	26.07	26.22	3.06	3.74	0.47	0.42	16.8	18.9	0.0364	0.4371	0.9	0.3857
2	8	0.3	0.3	-2.25	-0.5	70	-2.5	-2.5			-2.5	42.75	42.75	42.75	0	42	42.25	42.125	0.25	540	542	24.53	24.61	11.8	11.7	25	25.45	25.56	2.78	2.57	0.56	0.52	14.9	13.7	0.0368	0.4416	0.95	0.456
2	9	0.1	0.15	-2.75			-2.75	-2.5	-0.5	170	-2.75	43.25	44.5	43.875	1.25	43	44.5	43.75	1.5	607	591	24.75	24.79	11.2	11.3	29	25.34	25.28	3.31	2.96	0.56	0.51	15.1	15.9	0.0203	0.2441	0.49	0.2028
1	12	0.05	0.05	-3.75	-1.25	165	-4.375	-2.5	-1.5	180	-3.25	43.5	45.25	44.375	1.75	43.25	45.25	44.25	2	537	535	24.39	24.18	11.9	11.9	27	24.62	24.3	3.89	3.9	0.43	0.36	14.9	17.4	0.0085	0.1022	0.12	0.0533
1	7	0.3	0.3	-2.75	-0.75	180	-3.125	-2.75	-0.75	180	-3.125	43.75	45.25	44.5	1.5	43.75	45.25	44.5	1.5	509	511	24.41	24.35	12	12	32	25	24.92	4.64	4.14	0.5	0.51	13.3	16.4	0.0184	0.2213	0.57	0.2138
1	13	0.05	0.05	-3.25			-3.25	-2.75	-0.5	140	-3	42.75	43.25	43	0.5	42.5	43.25	42.875	0.75	556	583	24.71	25.54	11.4	11.3	27	24.76	24.69	3.42	3.59	0.48	0.4	19.8	19.3	0.0019	0.0222	-0.85	-0.378
1	11	0.05	0.05	-3			-3	-3.25			-3.25	44.75	45.25	45	0.5	44.25	45.25	44.75	1	534	526	24.18	24.35	11.4	11.4	28	24.83	25.12	3.37	3.04	0.6	0.58	20.5	17.5	0.0232	0.2786	0.77	0.33
2	10	0.2-	0.3+	-2.5			-2.5	-1.5	-0.5	170	-1.75	43.25	43.75	43.5	0.5	43.25	44.5	43.875	1.25	521	538	24.54	24.23	11.3	11.4	31	25.25	25.38	4.69	4.23	0.67	0.66	19.3	20.4	0.0229	0.2748	1.15	0.4452
1	9	0.03	0.03	-4.25	-0.5	160	-4.5	-4.5			-4.5	44.5	45.5	45	1	44.5	45.5	45	1	525	514	24.32	24.32	11.4	11.4	35	24.87	24.86	4.07	4.09	0.54	0.61	15.2	15	0.0157	0.1886	0.54	0.1851
1	15	0.05	0.05	-4.25			-4.25	-5	-0.75	180	-5.375	42.25	42.75	42.5	0.5	42.5	43.5	43	1	592	595	26.01	26.31	11.9	12	35	26.07	26.26	3.2	3.19	0.42	0.43	19	19	0.0017	0.0206	-0.05	-0.017
1	8	0.1	0.05	-2.75			-2.75	-3.5			-3.5	41.5	41.75	41.625	0.25	41.25	41.75	41.5	0.5	572	565	24.84	25.05	11.8	11.9	26	25.35	25.72	3.57	4.56	0.57	0.48	12	12	0.0196	0.2354	0.67	0.3092
2	9	0.1-	0.1-	-4	-0.5	170	-4.25	-3.5	-1	170	-4	41.75	42.75	42.25	1	41.5	42.75	42.125	1.25	561	565	25.78	25.81	11.8	11.8	31	26.57	26.41	3.55	3.49	0.52	0.52	17.4	13.4	0.0255	0.3058	0.6	0.2323

2	12	0.1	0.1-	-3.5	-0.75	5	-3.875	-3.75	-0.5	170	-4	42.25	43.5	42.875	1.25	42	43	42.5	1	572	567	25.3	25.57	12.1	12.2	22	25.48	25.63	2.99	3.15	0.61	0.52	18.8	20	0.0082	0.0982	0.06	0.0327
2	8	0.2	0.2	-2			-2	-2			-2	41.25	42.75	42	1.5	41.25	42.75	42	1.5	544	541	24.66	24.59	11.9	11.8	25	25.28	25.03	3.84	4.09	0.62	0.58	18.6	21.1	0.0248	0.2976	0.44	0.2112
2	7	0.2	0.2	-2.75	-1.25	180	-3.375	-2.75	-1.25	180	-3.375	40.5	42	41.25	1.5	41	42.5	41.75	1.5	587	570	25.02	24.79	11.5	11.6	16	25.64	25.36	3.75	3.74	0.53	0.53	12	13	0.0388	0.465	0.57	0.4275
2	11	0.05	0.05	-4			-4	-4			-4	42.25	42.75	42.5	0.5	42.5	43.5	43	1	566	562	25.88	25.72	11.9	11.9	28	25.8	25.81	3.52	3.2	0.51	0.53	17.8	20.4	-0.003	-0.034	0.09	0.0386
2	9	0.15	0.2	-2			-2	-1.75			-1.75	43.25	44.5	43.875	1.25	43	44	43.5	1	553	546	23.82	23.96	11.5	11.5	16	24.61	24.69	3.15	4.66	0.74	0.72	16	14	0.0494	0.5925	0.73	0.5475
1	9	0.2	0.3	-2.75			-2.75	-2.25			-2.25	42.75	44	43.375	1.25	42.75	44	43.375	1.25	559	555	24.81	24.49	11.9	11.9	29	25.11	24.7	4.34	3.55	0.53	0.51	16.6	15.5	0.0103	0.1241	0.21	0.0869
2	11	0.2	0.3	-2.75			-2.75	-2.5			-2.5	42	42.5	42.25	0.5	41.75	42.25	42	0.5	525	519	25.08	25.15	11.8	11.8	25	25.33	25.26	3.73	3.88	0.52	0.5	13.6	12.4	0.01	0.12	0.11	0.0528
1	9	0.05-	0.05-	-4			-4	-3.75			-3.75	42.25	43	42.625	0.75	41.75	42.75	42.25	1	600	592	24.63	24.65	11.9	11.8	29	24.79	24.71	5.99	4.94	0.59	0.53	16.4	14.4	0.0055	0.0662	0.06	0.0248
2	11	0.15	0.15	-4.75			-4.75	-4.5	-0.75	25	-4.875	44.5	44.75	44.625	0.25	44	44.75	44.375	0.75	524	508	25.31	25.36	11.4	11.4	31	25.78	25.59	4.47	4.24	0.57	0.5	14.9	14.3	0.0152	0.1819	0.23	0.089
1	9	0.3	0.3	-2			-2	-2.25			-2.25	41.75	42.25	42	0.5	42	42.5	42.25	0.5	594	583	24.41	24.5	11.4	11.4	30	24.15	24.71	3.62	3.71	0.64	0.6	19.2	21.1	-0.009	-0.104	0.21	0.084
2	8	0.4	0.4	-1.25			-1.25	-1.25	-0.5	170	-1.5	43	44	43.5	1	42.5	43.75	43.125	1.25	566	563	23.55	23.6	11.7	11.7	30	23.69	23.89	3.69	3.45	0.56	0.56	18	14.9	0.0047	0.056	0.29	0.116
2	9	0.4	0.4	-1.5			-1.5	-1.5			-1.5	42.5	43.5	43	1	42.5	43.25	42.875	0.75	564	560	23.64	23.77	11.9	11.9	31	23.87	24.13	4.13	4.81	0.53	0.54	17.5	15.2	0.0074	0.089	0.36	0.1394
1	14	0.1	0.1	-4.75	-0.5	155	-5	-5.25	-0.75	170	-5.625	43.5	45	44.25	1.5	43.25	44.75	44	1.5	578	577	25.79	25.99	11.6	11.7	26	25.99	26.06	3.65	3.66	0.31	0.45	21.3	22	0.0077	0.0923	0.07	0.0323
2	7	0.05	0.05	-4	-0.75	170	-4.375	-4.25	-0.75	180	-4.625	42.25	43.25	42.75	1	42.25	43.25	42.75	1	564	562	24.41	24.68	11.8	11.8	25	25.05	25.16	5.25	3.76	0.59	0.6	20	19.8	0.0256	0.3072	0.48	0.2304
1	10	0.2	0.2	-2.25			-2.25	-2			-2	41	41.75	41.375	0.75	40.75	41.5	41.125	0.75	574	577	24.84	24.83	11.8	11.7	21	25.01	25.12	3.73	3.93	0.68	0.58	14	16	0.0081	0.0971	0.29	0.1657
1	11	0.15	0.15	-3.25	-0.5	180	-3.5	-3	-1.25	180	-3.625	44.25	45	44.625	0.75	43.5	44.75	44.125	1.25	534	530	24.32	24.38	10.9	11.1	27	24.93	24.66	5.01	4.43	0.6	0.56	17.6	18.5	0.0226	0.2711	0.28	0.1244
1	9	0.1	0.2	-4	-0.5	180	-4.25	-3.25	-1	165	-3.75	41	42.25	41.625	1.25	40.75	42.25	41.5	1.5	576	566	25.83	25.77	11.7	11.7	24	26.48	26.7	4.6	5.22	0.63	0.53	14.3	12.8	0.0271	0.325	0.93	0.465
2	9	0.1	0.05	-3.25	-1	180	-3.75	-3.75	-1	170	-4.25	41.5	43	42.25	1.5	42	43.25	42.625	1.25	567	564	24.92	25.13	12	12	30	25.1	25.3	3.27	3.26	0.55	0.55	15.3	16.6	0.006	0.072	0.17	0.068
2	9	0.2	0.2	-2			-2	-1.75			-1.75	41.75	42.75	42.25	1	42	43.25	42.625	1.25	597	609	24.27	24.17	11.8	11.7	30	25.05	24.8	4.45	4.2	0.48	0.52	18.4	16	0.026	0.312	0.63	0.252
1	9	0.3-	0.3	-1.75			-1.75	-1.5			-1.5	41.75	42.75	42.25	1	41.75	42.75	42.25	1	566	550	23.69	23.69	11.5	11.6	26	23.81	24.25	3.58	4.34	0.56	0.53	15.9	13.5	0.0046	0.0554	0.56	0.2585
1	9	0.05-	0.05-	-4.5			-4.5	-4			-4	43	43.75	43.375	0.75	42.75	43.75	43.25	1	549	534	24.88	24.72	12	12	27	25.23	25.12	3.81	4.57	0.47	0.46	14.1	13.7	0.013	0.1556	0.4	0.1778
2	9	0.5	0.4	-1.5	-0.5	165	-1.75	-1.5	-0.5	170	-1.75	43	44	43.5	1	42.75	43.75	43.25	1	556	548	24.09	24.07	11.3	11.3	29	24.78	24.68	3.73	3.9	0.4	0.39	13	14	0.0238	0.2855	0.61	0.2524
2	8	0.05	0.05	-4	-0.5	40	-4.25	-3.5	-0.5	150	-3.75	43	44	43.5	1	43	44	43.5	1	543	540	24.82	24.58	11.7	11.7	24	25.78	25.64	4.45	2.99	0.53	0.53	14.9	16.2	0.04	0.48	1.06	0.53
1	11	0.3	0.2	-2			-2	-2.25			-2.25	42.5	42.75	42.625	0.25	42.5	42.75	42.625	0.25	556	551	24.99	25.27	11.9	11.9	28	25.19	25.38	3.01	3.27	0.31	0.35	20	18.8	0.0071	0.0857	0.11	0.0471
2	9	0.1	0.15	-3.25	-0.75	175	-3.625	-3.25	-0.75	180	-3.625	43	44.5	43.75	1.5	42.75	44.25	43.5	1.5	536	536	24.86	24.91	11.5	11.5	21	25.48	25.34	3.63	4.3	0.7	0.69	18.3	17.3	0.0295	0.3543	0.43	0.2457
2	9	0.15	0.15	-3	0	10	-3	-3	-0.5	170	-3.25	42.5	43.5	43	1	42.5	43.75	43.125	1.25	544	549	24.92	24.88	12	12	22	25.65	25.56	3.69	3.83	0.43	0.34	14.8	14.8	0.0332	0.3982	0.68	0.3709
1	9	0.2	0.2	-2.75			-2.75	-2.5			-2.5	44.25	44.5	44.375	0.25	44	44.5	44.25	0.5	551	536	23.66	23.53	11.7	11.7	14	24.12	24	3.65	3.06	0.38	0.42	16.7	18.6	0.0329	0.3943	0.47	0.4029
23	11	0.1	0.1	-3	-0.75	5	-3.375	-2.75	-1	165	-3.25	44	45.5	44.75	1.5	43.75	45.5	44.625	1.75	509	495	24.67	24.63	11.7	11.7	21	25.42	25.16	2.64	2.57	0.62	0.62	16.4	15.8	0.0357	0.4286	0.53	0.3029
2	9	0.15-	0.15	-2.75			-2.75	-2.5			-2.5	42.75	43.5	43.125	0.75	43	44	43.5	1	579	585	24.31	24.24	12	12	29	24.55	24.57	3.02	2.66	0.47	0.58	16.1	19	0.0083	0.0993	0.33	0.1366
1	9	0.15	0.15	-2.25			-2.25	-2.25			-2.25	41.25	42.25	41.75	1	41	41.75	41.375	0.75	498	490	24.74	24.82	12	12	25	25.11	25.29	4.75	3.99	0.49	0.58	10.2	10.9	0.0148	0.1776	0.47	0.2256
1	6	0.15	0.15	-1.75			-1.75	-1.75	-0.5	175	-2	43	44	43.5	1	43.5	44	43.75	0.5	552	541	23.84	23.81	12	12	23	24.99	24.83	3.51	4.61	0.45	0.39	12.9	9.1	0.05	0.6	1.02	0.5322
1	11	0.05	0.05	-3.75			-3.75	-3.75			-3.75	44.5	45	44.75	0.5	44	44.75	44.375	0.75	564	562	24.89	25	11	11	29	25.27	25.21	2.88	2.89	0.46	0.5	19.6	19.5	0.0131	0.1572	0.21	0.0869
1	8	0.1	0.1	-2.5	-0.75	150	-2.875	-2.5	-0.5	5	-2.75	44	45.25	44.625	1.25	44	45	44.5	1	560	552	23.39	23.52	11.3	11.3	27	24.25	24.3	3.36	3.2	0.56	0.61	14.5	16.2	0.0319	0.3822	0.78	0.3467
1	13	0.05	0.05	-5.25			-5.25	-5.5	-0.5	90	-5.75	45	45.75	45.375	0.75	44.75	45.25	45	0.5	545	533	23.79	24.09	11.1	11.1	13	23.72	23.85	3.6	3.51	0.57	0.48	13.8	13.8	-0.005	-0.065	-0.24	-0.222
2	6	0.2	0.2	-2			-2	-1.75			-1.75	44.25	45.5	44.875	1.25	44.25	45.25	44.75	1	548	545	23.75	23.68	11.2	11.3	15	24.4	24.3	3.33	3.26	0.4	0.37	14.1	16.8	0.0433	0.52	0.62	0.496
1	10	0.15	0.1	-3.75	-0.75	165	-4.125	-4.75	-0.75	165	-5.125	43.75	45.5	44.625	1.75	43.5	45.25	44.375	1.75	579	570	24.2	24.9	11.2	11.2	35	24.43	24.8	3.67	3.67	0.48	0.64	15.7	17.3	0.0066	0.0789	-0.1	-0.034
2	11	0.15	0.15	-3.5			-3.5	-3			-3	41.75	42.75	42.25	1	41.75	43	42.375	1.25	603	602	24.98	24.91	11.6	11.7	26	25.39	25.49	4.43	3.92	0.53	0.51	17.5	17.3	0.0158	0.1892	0.58	0.2677
1	8	0.2	0.2	-2.5			-2.5	-1.75	-1	180	-2.25	43	44.25	43.625	1.25	42.75	44.5	43.625	1.75	554	565	24.84	24.64	12.3	12.3	32	25.96	25.65	4.05	3.69	0.46	0.57	17.4	13.7	0.035	0.42	1.01	0.3787
1	12	0.5	0.25	-1.25	-1	170	-1.75	-2.25	-1.25	175	-2.875	43	44.5	43.75	1.5	42.75	44	43.375	1.25	558	559	24.92	24.44	12.3	12.3	25	24.05	24.15	3.36	3.85	0.23	0.24	14.8	10.7	0.0012	0.0144	-0.29	-0.139
1	8	0.2	0.2	-3			-3	-2.25	-1	175	-2.75	42.5	44	43.25	1.5	42.5	44.5	43.5	2	587	576	24.19	24	11.3	11.4	21	24.66	24.51	3.94	4.12	0.71	0.61	20.4	21.8	0.0224	0.2686	0.51	0.2914
1	10	0.3	0.3	-1.75			-1.75	-1.75			-1.75	42.75	43.75	43.25	1	42.75	43.75	43.25	1	578	563	24.45	24.21	11.7	11.7	13	24.78	24.42	4.5	4.14	0.52	0.53	19	22.8	0.0254	0.3046	0.21	0.1938
2	10	0.05	0.05	-5			-5	-5			-5	44.25	45.5	44.875	1.25	44.25	45.75	45	1.5	527	522	24.43	24.33	11.6	11.7	26	24.77	24.56	3.97	4.4	0.53	0.53	13.5	13.3	0.0131	0.1569	0.23	0.1062
1	10	0.15	0.15	-4	-3.25		-5.625	-4			-4	44.75	45.25	45	0.5	44.5	45.5	45	1	552	564	24.53	24.54	11.6	11.6	21	24.86	24.8	3.85	4.39	0.45	0.46	19	18.1	0.0157	0.1886	0.26	0.1486
2	11	0.12	0.15	-4.5	-1.25	180	-5.125	-4	-1.5	180	-4.75	43.5	45	44.25	1.5	43.5	45.25	44.375	1.75	485	487	25.96	25.55	12	12	30	25.73	25.61	3.42	3.18	0.45	0.57	15.1	14.4	-0.008	-0.092	0.06	0.024
2	13	0.5	0.5	-1.5			-1.5	-1			-1	42.75	43.5	43.125	0.75	43.25	44	43.625	0.75	554	547	25.14	24.6	12	12	21	25.2	24.98	3.92	3.73	0.4	0.44	13.2	16.6	0.0029	0.0343	0.38	0.2171
1	9	0.2	0.2	-3			-3	-2.75			-2.75	42.75	43.75	43.25	1	43	43.75	43.375	0.75	507	515	24.24	24.08	11.8	11.8	22	24.9	24.58	3.47	3.64	0.72	0.75	12.9	13.5	0.03	0.36	0.5	0.2727
2	7	0.1	0.1	-4			-4	-4.25			-4.25	44.5	45	44.75	0.5	44	45	44.5	1	570	564	24.45	24.59	11.7	11.7	28	25.76	25.78	3.59	3.36	0.4	0.37	16.2	18.4	0.0468	0.5614	1.19	0.51
1	8	0.3	0.3	-2.25			-2.25	-2.25			-2.25	43	44.75	43.875	1.75	43.5	44.75	44.125	1.25	545	559	24.1	24.02	11.9	12	25	24.32	24.33	4.03	4.19	0.34	0.51	14.4	12.9	0.0088	0.1056	0.31	0.1488
2	8	0.3	0.3	-2			-2	-2			-2	42	43	42.5	1	41.75	42.5	42.125	0.75	559	575	24.32	24.61	11.9	11.8	23	24.98	25.3	4.28	4.4	0.52	0.43	18.9	20.8	0.0287	0.3443	0.69	0.36
1	12	0.25	0.25	-2.5	-0.5	10	-2.75	-2	-0.5	170	-2.25	42.75	43.75	43.25	1	42.75	44	43.375	1.25	548	536	25.05	24.82	11.7	11.7	29	25.02	24.59	4.42	5.36	0.74	0.67	16.8	15.5	-0.001	-0.012	-0.23	-0.095
1	8	0.15	0.15	-3			-3	-3.25			-3.25	43.5	43.75	43.625	0.25	43.5	43.5	43.5	0	550	541	24.46	24.47	11.5	11.4	25	25.3	25.19	2.96	3.36	0.31	0.15	15.4	17	0.0336	0.4032	0.72	0.3456
1	10	0.1	0.1	-4.5	-0.5	130	-4.75	-4.75			-4.75	43.5	44.75	44.125	1.25	44	45	44.5	1	549	545	24.86	24.76	11.8	11.7	19	25.32	25.33	2.99	3.26	0.49	0.6	14.5	13.6	0.0242	0.2905	0.57	0.36
1	8	0.1	0.1	-3.5			-3.5	-3.5			-3.5	42.75	43.25	43	0.5	42.75	43.25	43	0.5	530	542	25.01	24.97	11.8	11.8	25	25.57	25.51	3.31	3.94	0.53	0.55	21	21	0.0224	0.2688	0.54	0.2592
2	8	0.3	0.5	-1.75	-0.5	180	-2	-0.75	-1	175	-1.25	43.5	45	44.25	1.5	43.5	45.25	44.375	1.75	557	550	24.04	23.68	12	12	21	24.65	24.32	4.8	4.2	0.58	0.41	16.5	18.2	0.029	0.3486	0.64	0.3657
1	13	0.5	0.3	-3			-3		-0.75	180	-0.375	43.25	44.5	43.875	1.25	42.5	44.25	43.375	1.75	595	585	24.38	24.81	11.8	11.8	21	24.27	24.67	4.05	4.1	0.5	0.27	18.6	18.5	-0.005	-0.063	-0.14	-0.08
1	11	0.1+	0.1	-3.5			-3.5	-3.75	-1.5	170	-4.5	42	42.75	42.375	0.75	41.5	43.25	42.375	1.75	558	565	25.32	25.8	12.1	12.1	23	25.59	25.75	3.8	5.33	0.49	0.47	15.3	14.9	0.0117	0.1409	-0.05	-0.026
1	12	0.4-	0.3	-2.25			-2.25	-2	-0.5	160	-2.25	44.25	44.75	44.5	0.5	44	45	44.5	1	544	546	23.59	23.59	11.6	11.6	23	23.73	23.8	4.01	4.4	0.5	0.41	17.1	16.8	0.0061	0.073	0.21	0.1096
2	8	0.2	0.15	-1			-1	-1	-0.5	10	-1.25	40.5	41.5	41	1	40.75	41.75	41.25	1	573	557	23.9	23.9	12.1	12.1	24	24.41	24.5	4.51	4.36	0.46	0.38	13.9	15.1	0.0213	0.255	0.6	0.3
1	8	0.1	0.1	-3	-1.75	175	-3.875	-3	-0.75	180	-3.375	44.25	45.75	45	1.5	44	45.5	44.75	1.5	483	487	23.26	23.34	12.1	12.1	34	23.47	23.61	4.1	3.67	0.45	0.47	18.6	15.9	0.0062	0.0741	0.27	0.0953
2	10	0.2	0.2	-2.75	-0.5	160	-3	-2	-1.25	175	-2.625	41.5	42.5	42	1	41	42.75	41.875	1.75	579	567	25.46	25.35	12.1	12.1	26	26.2	26.12	4.3	4.2	0.5	0.5	16	17	0.0285	0.3415	0.77	0.3554
	9.747	0.1633	0.1758	-2.897	-0.706		-3.033	-2.797	-0.754		-2.983	42.77	43.784	43.277	1.0141	42.708	43.825	43.267	1.1175	553.99	552.41	24.623	24.62	11.692	11.695	29.618	25.125	25.103	3.8711	3.8753	0.5378	0.541	15.687	15.583		0.2079		
	1.9624	0.1185	0.1246	1.0555	0.377		1.1119	1.1313	0.3097		1.1808	1.0212	1.1087	1.0448	0.4216	1.0216	1.1337	1.0556	0.4485	30.451	30.277	0.6717	0.7075	0.3154	0.3077	6.642	0.6841	0.6968	0.5569	0.588	0.1216	0.1153	2.6915	3.1939		0.1509		
